# Comparative responsiveness of shoulder patient-reported outcome measures (PROMs) to rotator cuff repair surgery and healing

**DOI:** 10.1016/j.jse.2026.01.010

**Published:** 2026-02-18

**Authors:** Sambit Sahoo, Yadi Li, Charles J. Cogan, Vahid Entezari, Jason C. Ho, Joseph P. Iannotti, Eric T. Ricchetti, Brittany Lapin, Kathleen A. Derwin

**Affiliations:** a Department of Biomedical Engineering, Cleveland Clinic, Cleveland, OH, USA; b Department of Orthopaedic Surgery, Cleveland Clinic, Cleveland, OH, USA; c Cleveland Clinic Lerner College of Medicine of Case Western Reserve, University School of Medicine, Cleveland Clinic, Cleveland, OH, USA; d Department of Quantitative Health Sciences, Cleveland Clinic, Cleveland, OH, USA

**Keywords:** Shoulder, rotator cuff repair, structural integrity, tendon healing, patient reported outcomes, PROMs

## Abstract

**Background:**

Patient-reported outcome measures (PROMs) are routinely used to assess pain, function, and quality of life in shoulder care. Although rotator cuff repair (RCR) is a highly effective treatment for symptomatic tears, the relationship between structural healing and PROM responsiveness remains unclear. This study aimed to evaluate and compare the responsiveness of 5 common shoulder PROMs—the Penn Shoulder Score, modified American Shoulder and Elbow Surgeons score, Single Assessment Numeric Evaluation, Shoulder Activity Level (SAL), and Patient-Reported Outcome Measure Information System Upper Extremity—as well as individual items from these measures and the Western Ontario Rotator Cuff Index, to RCR surgery and healing at 1 year postoperatively, with secondary analyses at 6 months and 2 years.

**Methods:**

A prospective cohort of 117 patients undergoing arthroscopic RCR for fully reparable 1–5 cm supraspinatus/infraspinatus tears was analyzed. PROMs were collected preoperatively and at 6 months, 1 year, and 2 years postoperatively. RCR healing was assessed using magnetic resonance imaging-based Sugaya classification and computed tomography-measured tendon retraction. Responsiveness was evaluated using standardized response means, with subgroup analyses comparing healed and nonhealed patients. Correlations between PROMs and structural healing were analyzed.

**Results:**

All PROMs and their individual items (except SAL) demonstrated high responsiveness to RCR surgery (standardized response mean >0.8) during the first 2 postoperative years, regardless of structural healing status, with the majority of gains occurring within the first 6 months. However, neither total PROMs nor select high-function items demonstrated correlations with structural healing (r < 0.3), indicating PROMs improvements primarily reflected reduced pain and enhanced daily function rather than RCR integrity. At 1 year, 92% of patients reported an acceptable symptom state (Patient Acceptable Symptom State “yes”), including all patients meeting stringent criteria for failed RCR. SAL was unresponsive to RCR surgery in the overall cohort and demonstrated limited utility in assessing functional differences in this patient population. Penn Shoulder Score, American Shoulder and Elbow Surgeons, Single Assessment Numeric Evaluation, and Patient-Reported Outcome Measure Information System Upper Extremity demonstrated progressively increasing ceiling effects postoperatively.

**Conclusion:**

Shoulder PROMs are highly responsive to RCR surgery but even their highest function items lack sensitivity to structural healing during the first 2 postoperative years. PROM improvements primarily reflect subjective gains in pain relief and daily function, highlighting the need for alternate outcome measures incorporating objective functional assessments to better define the impact of RCR healing in the early term. Future research should focus on developing PROMs with lesser ceiling effects postoperatively and evaluating the longer-term clinical consequences of failed structural RCR healing.

**Level of evidence:**

Basic Science Study; Validation of Outcome Instruments

Patient-reported outcome measures (PROMs) are essential tools in health care to monitor patient outcomes, evaluate treatment efficacy, and inform clinical decision-making. In orthopedics, particularly in shoulder care, several validated shoulder-specific PROMs are widely used to assess shoulder function, pain, and quality of life following various shoulder pathologies and surgeries. Among these, the American Shoulder and Elbow Surgeons (ASES) score is the most commonly used.^13,40^ Other frequently used shoulder-specific PROMs include the Penn Shoulder Score (PSS),^33^ Single Assessment Numeric Evaluation (SANE),^43^ Shoulder Activity Level (SAL),^5^ and the Western Ontario Rotator Cuff Index (WORC).^18^ More recently, Patient-Reported Outcome Measure Information System Upper Extremity (PROMIS-UE),^27^ which is based on computer adaptive testing, has been introduced as a more efficient method for assessing upper extremity function. These PROMs have been validated for a variety of shoulder conditions, providing clinicians with valuable tools to assess outcomes and guide treatment strategies.

Rotator cuff tendon tears are among the most common shoulder pathologies, with many patients requiring surgery when nonoperative treatments fail. Rotator cuff repair (RCR) is considered a highly effective surgery, as most patients experience significant improvement in PROMs postoperation.^34^ However, studies have shown that at short-term follow-up, PROM scores often do not differ significantly between patients with anatomically healed repairs and those who experience retears,^26^ whereas objective shoulder function—including range of motion, strength, and the ability to use the arm away from the body and overhead—is significantly better when the repaired tendon remains intact.^12^ To date, limited research has compared the responsiveness of different shoulder-specific PROMs after RCR—defined as their ability to detect clinically meaningful changes in a patient’s health status following treatment—or examined whether individual items within these instruments are particularly sensitive to healing-related changes.^4,10,36,42^ For example, certain PROM items reflecting higher-level shoulder function may show responsiveness to healing, even when overall PROM scores do not distinguish between patients who heal and those who do not.

The primary aim of this study was to evaluate and compare the responsiveness of several common shoulder PROMs (PSS, modified ASES, PROMIS-UE, SAL, and SANE) as well as individual items from these measures and the WORC to RCR surgery and healing at 1-year postoperation. We hypothesized that patients who healed would show excellent responsiveness across all PROMs and individual items, whereas those who did not heal would exhibit poor responsiveness in items and measures associated with higher shoulder function (eg, overhead activities or weightlifting). As a secondary objective, we performed our analyses at 6-month and 2-year postoperatively to investigate responsiveness of these PROMs and individual items to structural healing at different time points.

## Methods

### Rotator cuff repair surgical cohort

A total of 117 patients undergoing primary arthroscopic RCR by one of 7 shoulder surgeons at our institution between 2016 and 2021 were enrolled into a prospective cohort study (IRB 16–089, ClinicalTrials.gov# NCT02716441). Eligible patients were aged 18–75 years and had 1–5 cm tears of the supraspinatus and/or infraspinatus tendons that were fully reparable using an arthroscopic double-row technique. Patients were excluded if they required repair of the subscapularis tendon or labral tears, had advanced glenohumeral arthritis (Outerbridge Grade 3 or 4), or had prior RCR or shoulder arthroplasty. All RCR procedures were performed according to the surgeons’ preferred arthroscopic double-row techniques (eg, conventional, suture bridge, and speed bridge).^2^ Following repair, two-to-four radiopaque tissue markers (FibermarX, Viscus Biologics LLC, Cleveland, OH) were sutured to the superficial surface of the tendon near the repair site to enable measurement of tendon retraction.^17,31^

### Patient-reported outcomes

Patients completed PSS, modified ASES score, SANE, SAL, PROMIS-UE, and various items from WORC preoperatively and postoperatively at 6 months, 1 year, and 2 years.

PSS includes 3 pain questions, 20 function questions, and a question related to satisfaction.^22^ PSS is scored from 0 to 100 points (pain subscore: 0–30, function subscore: 0–60, satisfaction subscore: 0–10 points), with higher scores representing less pain, better function, and higher satisfaction.^22^ The modified ASES score includes 1 question for pain and 10 of the 20 PSS function questions and is scored from 0 to 100 (pain subscore: 0–50, function subscore: 0–50 points), with higher scores representing less pain and better function.^13 ,32^ The 10 function items unique to PSS were introduced to capture a broader spectrum of shoulder function and pain than the 10 function items common with ASES.^22^ These additional items span low-load, waist-level tasks (eg, f3, f9, and f11) to high-load, overhead activities (eg, f13, f16, and f19) (Supplementary Table S1). Similarly, the three PSS pain items interrogate pain levels encountered during rest, normal daily activities, or with strenuous movements, whereas the ASES pain question simply asks the patient, “How bad is your pain today?”

SANE asks a single question to assess shoulder status, “How would you rate your shoulder today as a percentage of normal (0% - 100% scale with 100% being normal)?”^40,43^ SAL is scored from 0 to 20 points based on the frequency of performing 5 activities ranging from carrying objects 8 pounds or heavier to lifting objects 25 pounds or heavier.^5^ PROMIS-UE is administered as a computer adaptive test that derives from a 16 question item bank (version 1.2) and has been shown to be valid, reliable, easy to use, and more efficient than legacy shoulder PROMs.^27^ Seven items from work and lifestyle domains of the WORC^18^ were also asked (Supplementary Table S1). Finally, Patient Acceptable Symptom State (PASS) was assessed at 1 year using a yes/no response to whether patients considered their shoulder condition satisfactory, based on daily activity, pain, and functional limitations (“Taking into account all the activity you have during your daily life, your level of pain, and also your activity limitations and participation restrictions, do you consider the current state of your shoulder satisfactory?”).^8^

### RCR integrity (Sugaya classification from magnetic resonance imaging)

Patients had a shoulder magnetic resonance imaging performed at 6 months, 1 year, and 2 years, following RCR on 1.5 T scanners (Siemens Medical Solutions) using a dedicated shoulder coil while lying in a supine position with the arm at the side.^23,24^ Two musculoskeletal radiologists, blinded to PROMs and tendon retraction data, reviewed the deidentified magnetic resonance imagings independently and in random order using prospectively defined reading rules. The superior-posterior RCR site was assigned a Sugaya classification, a standard clinical grading of tendon healing following RCR, with **1** = normal, **2** = intact attenuated, **3** = partial-thickness tear, **4** = small, and **5** = large full-thickness tear.^37,38^ The radiologists’ Sugaya grades were compared for clinically significant discrepancies (ie, 1 or 2 vs. 3, 4 or 5; 3 vs. 4 or 5). Scans with such discrepancies were re-examined jointly by the readers and assigned a consensus Sugaya grade.^23^ For discrepancies deemed clinically less significant (eg, 1 vs. 2 or 4 vs. 5), Sugaya grades between readers were averaged.

### RCR tendon retraction (radiopaque markers by computed tomography imaging)

Patients had a low-dose shoulder computed tomography (CT) scan (100–120 kV, 45–85 mAs) performed on the day of surgery and at 6 months, 1 year, and 2 years following RCR using 16 to 64 channels systems (Siemens Medical Solutions) with isotropic voxels ranging from 0.6 to 0.75 mm. CT scans were obtained with patients in a standardized supine position with the arm at their side.^17^ Tendon retraction was quantified at each postoperative time by measuring the change in distance (mm) from the radiopaque markers to the humeral greater tuberosity on CT images,^9,25,31^ where apparent tendon retraction of >6.7 mm exceeds the usual range of its measurement variation.^17^

### RCR healing

‘RCR healing’ was defined in 2 ways. First, we used the conventional dichotomous Sugaya classification where Sugaya 1/2/3 was considered “healed” and Sugaya 4/5 was considered “not healed.”^38^ Second, because significant tendon retraction in the absence of a recurrent tendon defect (so-called “failure with continuity”) can mask apparent failed repairs,^25^ we also investigated a more stringent definition that combined Sugaya classification with CT-based tendon retraction measurements described above. Under this definition, Sugaya 1/2/3 repairs were even more stringently classified as ‘healed’ if associated with ≤10 mm tendon retraction and as ‘not healed’ if Sugaya 4/5 *with* ≥15 mm tendon retraction; patients not meeting either criterion were excluded from this analysis. These tendon retraction thresholds have not been previously validated but were defined *a priori* and informed by the reported mediolateral dimension of the superior rotator cuff tendon footprint (~10 mm).^11,35^ Accordingly, the ≤10-mm threshold was intended to reflect tendon retraction within the approximate footprint dimension, whereas the ≥15-mm threshold represented retraction clearly exceeding this dimension. Healing status was determined at 6 months, 1 year, and 2 years using observed data or mean of imputed values at the respective time point.

## Statistical analysis

### Summary statistics

Demographics, rotator cuff tear characteristics, PROM summary scores and individual items, and healing measures (Sugaya and tendon retraction) were summarized by mean with standard deviation for continuous variables and count with percentage for categorical variables. Continuous data were assessed for normality using histograms and q-q plots. Floor and ceiling effects for PROMs were calculated as the percentage of respondents reporting the lowest and highest possible scores, respectively. Floor and ceiling effects <15% are considered acceptable.^39^

### Missing data

A total of 113 patients who completed the 2-year follow-up were included in this analysis. Data on demographics, clinical characteristics, and PROMs total and subscores were complete in these 113 patients. Sugaya was missing for 2 patients at 2-year follow-up, and some tendon retraction data were missing for individual markers in 28 patients at 6 months, 33 at 1 year, and 38 at 2 years. Missing data in Sugaya and tendon retraction were assumed to be missing at random and multiply imputed using multivariate imputation by chained equations (mice R package^41^), with 50 imputations, from a comprehensive set of variables at baseline and follow-up time-points. Missingness was primarily encountered in tendon retraction data due to dislodged individual tendon markers that had detached from their initial location at various time-points and were no longer visualizable or appeared in anomalous locations. Since numbers and placement of retraction markers vary across participants, pattern mixture multiple imputation was performed in subsets of the cohort determined by marker placement patterns. Tendon retraction measurements from all markers in each patient, including those derived by imputation, were averaged to provide a mean tendon retraction value at each time point. Results were pooled from imputed datasets using Rubin’s standard formula.^29^

### Association between PROMs and RCR healing

Associations between PROM summary scores and individual items with measures of healing at 6 months, 1 year, and 2 years were evaluated using Spearman correlation coefficients.

### Responsiveness of PROMs and individual items to RCR surgery and RCR healing

To assess the responsiveness of PROMs and individual items to RCR surgery and healing, changes in PROM summary scores and individual items from preoperative to 6-month, 1-year, and 2-year follow-up were assessed in the overall cohort and compared between the healed and not-healed groups. Paired t-tests were used to assess changes from preoperative to each follow-up time point. Standardized response means (SRMs) were calculated as the mean change divided by its standard deviation. SRM values were interpreted as: poor (≤0.40), fair (0.41–0.59), good (0.60–0.75), and excellent (≥0.75) responsiveness.^6,32^

Aside from multiple imputation, all analyses were conducted using SAS Enterprise Guide version 8.2 (SAS Institute, Cary, NC). Statistical significance was established throughout at *P* < .05; however, interpretations were primarily based on magnitudes of effects.

## Results

### Demographic and surgical characteristics

Three patients were lost to follow-up, including one who had died. Additionally, 1 patient underwent a reoperation between 6 months and 1 year after RCR. A total of 113 patients completed the 2-year follow-up and were included in this analysis. Patients averaged 58.5 ± 8.5 years of age with a body mass index of 29.9 ± 5.9 (Table I). The majority were White (86.7%), male (56.6%), and employed (61.1%). A total of 8.8% were current smokers, and 36.3% were former smokers. Rotator cuff tear dimensions averaged to 2.2 ± 0.86 cm antero-posteriorly and 1.2 ± 0.52 cm medio-laterally based on intraoperative measurement at the time of RCR. Fifty-four percent had a tenodesis of the long head of biceps, and 65% had an acromioplasty.

### PROMs

**Figure 1 –:**
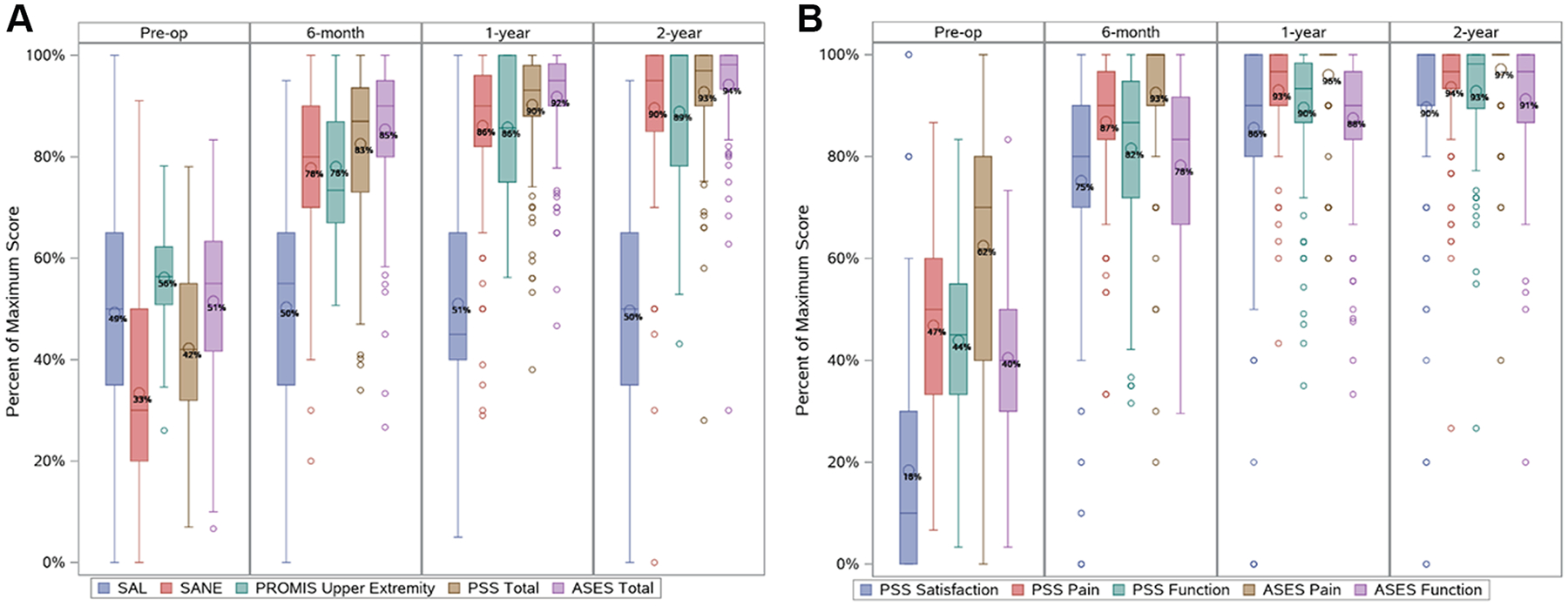
Box plots of (**A**) PROMs and (**B**) PROM subscores expressed as a percentage of maximum score. Maximum scores are as follows: SAL (20), SANE (100), PROMIS-UE (56.4), PSS-Total (100), ASES-Total (100), PSS-Satisfaction (10), PSS-Pain (30), PSS-Function (60), ASES-Pain (50), and ASES-Function (50). *SANE*, Single Assessment Numeric Evaluation; *SAL*, Shoulder Activity Level; *PSS*, Penn Shoulder Score; *ASES*, American Shoulder and Elbow Surgeons; *PROM*, patient-reported outcome measure; *PROMIS-UE*, Patient-Reported Outcome Measure Information System Upper Extremity.

The mean (standard deviation) of the 5 PROMs and any respective subscores before and after RCR are provided in Table II and by item in Supplementary Table S2. All PROMs and their items improved postoperatively, except SAL, with most of the improvement evident by 6 months and a more gradual increase thereafter. PROMs and their subscores are shown graphically as a percentage of their maximum in Fig. 1. PSS, modified ASES, SANE, and PROMIS-UE demonstrated a progressively increasing ceiling effect postoperatively (Fig. 1A, Table II). PSS-Satisfaction showed a significant floor effect at baseline, and the subscores of PSS and ASES followed a similar pattern of postopertative improvement as the total scores (Fig. 1B, Table II). At 1 year, 104 of 113 patients (92%) reported a satisfactory shoulder state (PASS “yes”).

### Sugaya classification, tendon retraction as measures of RCR healing

**Figure 2 –:**
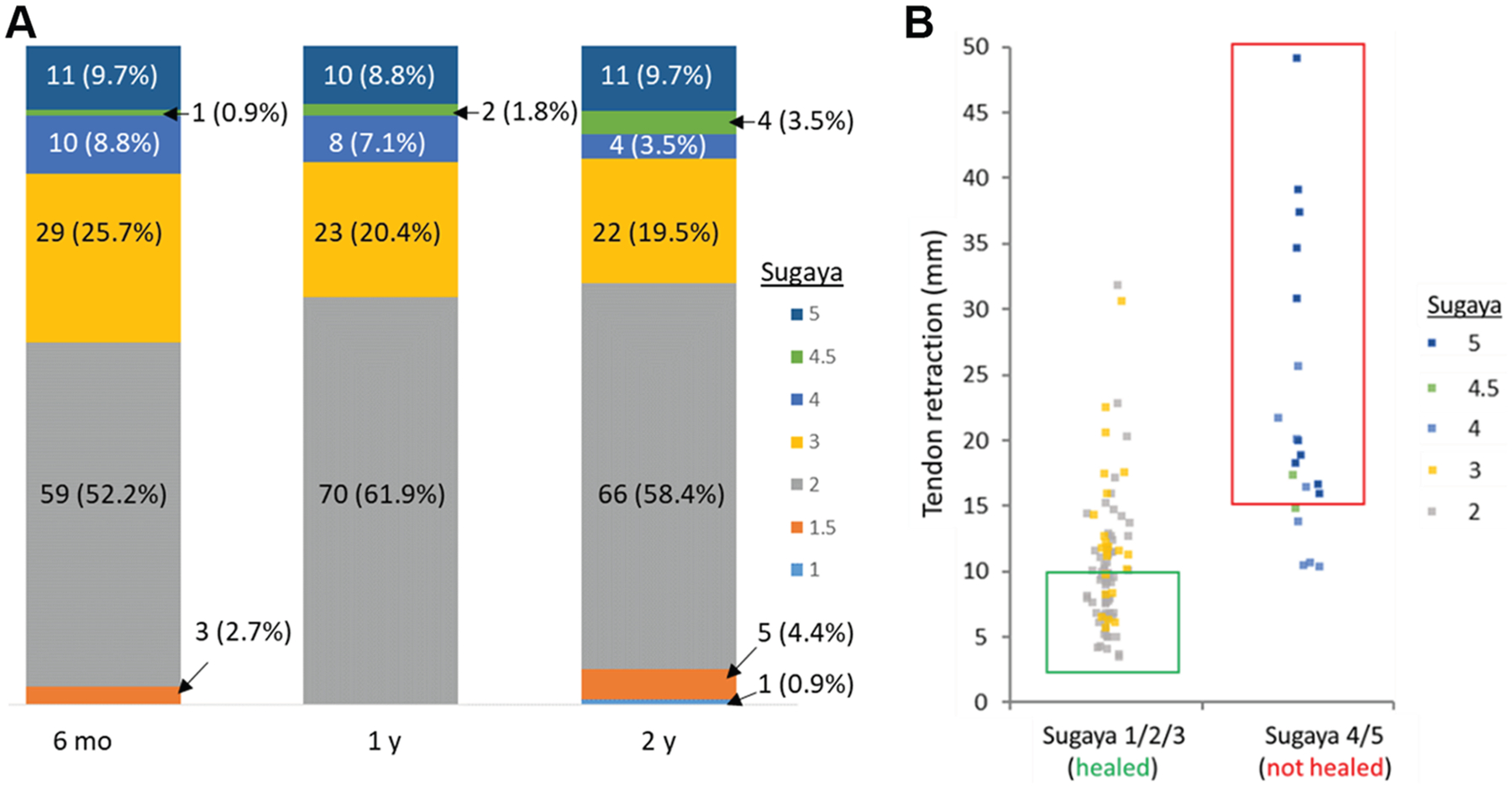
(**A**) RCR healing status at 6 months, 1 year, and 2 years based on MRI-based Sugaya classification of patients. (**B**) Distribution of CT-based tendon retraction data at 1 year, grouped according to the conventional dichotomous Sugaya classification, where 1/2/3 are considered “healed” and 4/5 “not healed.” Boxes delineate the patients who met the more stringent RCR healing definition that relies on both Sugaya score and tendon retraction. Imputation datasets accounting for missing data in Sugaya and tendon retraction were used. *RCR*, rotator cuff repair; *CT*, computed tomography; *MRI*, magnetic resonance imaging.

Seventeen to nineteen percent of patients had a Sugaya 4/5 (recurrent defect) between 6 months and 2 years following RCR (Fig. 2A), and average tendon retraction ranged from 12 ± 7 mm to 13 ± 7 mm during this period (Supplementary Table S3). Patients with Sugaya 4/5 averaged appreciably more tendon retraction than those with Sugaya 1/2/3 at all 3 time points; however, retraction varied considerably even in the Sugaya 1/2/3 patients without a recurrent defect (eg, range, 3.5 to 31.8 mm at 1 year) (Fig. 2B).

Using the conventional RCR healing definition that relies only on Sugaya classification, 93 (82%) patients were considered to have a healed RCR and 20 (18%) to have a failed RCR at 1 year (Table III). Using the more stringent RCR healing definition that relies on both Sugaya score and tendon retraction, 47 (42%) patients were considered to have a healed RCR and 15 (13%) to have a failed RCR at 1 year, with 51 (45%) patients not meeting either criterion. The fraction of patients assigned to each healing group at 6 months and 2 years varied slightly and is shown in Table III.

### Association between PROMs and RCR healing

The correlation between PROMs and RCR healing measured by either Sugaya classification or tendon retraction was weak to nonexistent (r < 0.3) at all time points (Table IV, Supplementary Table S4). Similarly, the correlation between all individual PROM items and Sugaya classification or tendon retraction was weak to nonexistent at all time points (Supplementary Table S5). In the few instances where statistical significance was demonstrated, these weak correlations were in the opposite direction of that hypothesized; better PROMs were associated with worse healing. All 15 patients who met the stringent criteria for failed RCR at 1 year reported a satisfactory symptom state (PASS “yes”). Conversely, none of the 9 patients who reported an unsatisfactory symptom state (PASS “no”) met the stringent criteria for failed RCR.

### Responsiveness of PROMs and individual items to RCR surgery and RCR healing

Paired analysis of preoperative and postoperative PROMs showed that, on average, patients reported substantive improvement in pain, function, and satisfaction after RCR, indicating a high level of responsiveness (SRM >0.8) to RCR surgery at all time points (Tables V, Supplementary Tables S6–S8). SAL was the only PROM that was not responsive to RCR surgery at the overall cohort level.

Subgroup analysis of patients with healed and nonhealed repairs according to the conventional definition of RCR healing, showed high levels of responsiveness for all PROMs except SAL in both groups and at all time points, suggesting that responsiveness was not dependent on the “healing” status (Tables V, Supplementary Tables S6–S8). At the item level, every item in each PROM was highly responsive at all time points regardless of healing status, except the SAL items which were generally unresponsive (Supplementary Tables S6–S8).

Sensitivity analysis using the more stringent RCR healing definition similarly showed that patients with both healed and nonhealed repairs exhibited high responsiveness across all PROMs and their individual items at all time points, except for SAL (Supplementary Tables S9–S11). While individual SAL items were not responsive to this stringent healing definition, overall SAL scores were. Notably, the nonhealed subgroup showed a significant increase in overall SAL scores at all postoperative time points, indicating an increase in activity level, whereas the healed group did not.

## Discussion

The primary aim of this study was to evaluate and compare the responsiveness of various shoulder PROMs―PSS, modified ASES, PROMIS-UE, SAL, and SANE―as well as individual items from these instruments and the WORC, to RCR surgery and repair healing at 1 year postoperatively. Overall, all PROMs and their individual items (except SAL) demonstrated high responsiveness to RCR during the first 2 postoperative years, regardless of structural healing status, with the majority of gains occurring within the first 6 months.

We initially hypothesized that patients with RCR healing would show excellent responsiveness across all PROMs and individual items, whereas those who did not heal would exhibit poor responsiveness in measures associated with higher shoulder function. In contrast, nearly all PROMs were highly responsive regardless of healing status, whether RCR healing was defined using a conventional definition (Sugaya grades 1–3) or stringent criteria (Sugaya grades 1–3 and ≤1 cm of tendon retraction). Correlation analysis further confirmed weak to nonexistent correlations (r < 0.3) between structural healing and all PROMs and individual items across all time points. At 1 year, all patients who met the stringent criteria for failed RCR still reported an acceptable state (PASS “yes”), while no patient who reported an unacceptable state (PASS “no”) had a structurally failed RCR. These findings demonstrate a lack of congruency between structural healing and better 1 year outcomes from the patient’s perspective.

Among the evaluated PROMs, SAL was the sole outlier. Its low and unchanged scores (~10 of 20 points) throughout follow-up suggests that most patients in this cohort infrequently engaged in the high-demand shoulder activities assessed by SAL. This indicates that SAL may be more appropriate for younger or athletic populations but lacks sensitivity to detect meaningful functional differences in older, lower-demand patients typically undergoing RCR. Interestingly, a modest yet statistically significant improvement in SAL scores (~3 point increase) was observed only in the nonhealed group (as defined by the stringent criteria) across all postoperative time points. This improvement likely does not reflect functional benefits of nonhealing but may instead signal a premature return to higher activity levels—potentially overloading the repair site leading to structural failure. This interpretation aligns with prior research, which suggests that early postoperative activity restriction and stiffness can be protective against RCR failure, whereas increased shoulder use may compromise repair integrity, even if patients report subjective functional improvement.^14,19^ These findings highlight the complexity of interpreting activity-based PROMs like SAL in lower-demand populations and underscore the need to consider postoperative behavior and rehabilitation adherence when evaluating surgical outcomes.

At the item level, most PROM items showed substantial improvement after surgery, regardless of healing status—again, with SAL items as the exception. In general, function items assessing strenuous activities, such as f13, f16, and f19, exhibited higher responsiveness (SRMs: 1.7–2.18 at 1 year). These items are associated with activities that require high loads at or above the shoulder level. Conversely, items related to lighter tasks, which involve lower loads at or below the waist level (eg, f3, f9, and f11) displayed lower responsiveness (SRMs: 1.08–1.42 at 1 year). Similarly, PSS pain during strenuous activities showed higher responsiveness (SRM: −3.1 at 1 year) compared to PSS pain during rest or normal activities (SRMs: −1.1 to −1.9 at 1 year).

Collectively, these findings demonstrate that none of the shoulder PROMs evaluated, nor their individual items, were able to detect a benefit of structural tendon healing in this cohort, at least during the early postoperative period. At the total PROM level, these findings are consistent with prior reports.^26,30^ For example, a meta-analysis of 5 level I-II studies found no significant differences in postoperative ASES scores between patients with intact repairs and those with retears.^30^ No prior reports on item level responsiveness to RCR healing are available for comparison, but our findings were not consistent with our hypothesis that a few higher-function items would be responsive to RCR healing, even if the total PROM was not.

As we consider the implications of our findings, it is important to note that most PROMs were originally designed to assess baseline symptoms rather than to measure changes postintervention, and as such are subject to ceiling effects postoperatively.^3,10,16,21^ In this study, SANE and PSS-Total showed the lowest ceiling effects—likely due to the finer granularity of SANE’s 0–100 scale and the more comprehensive assessment of function and pain by PSS. Ceiling effects limit a PROM’s ability to detect continued improvement beyond a certain threshold, limiting their longitudinal validity and possibly obscuring subtle differences in healing outcomes beyond the 6-month postoperative time-point. Further, improvement in PROMs postintervention is subject to bias from both placebo and exaggeration of treatment effects that are often seen following invasive treatments.^20^

Despite the inherent limitations of PROMs as an outcome tool, our findings meaningfully inform the ongoing clinical debate regarding relevance of tendon integrity following RCR. Using a stringent definition of structural healing based on the absence of a recurrent defect and limited tendon retraction, we found that total PROMs were highly responsive to RCR surgery but not to healing in the early postoperative period. Importantly, and for the first time, we also showed these findings persisted even when analysis was restricted to individual high-function items. Together, our results suggest that PROMs in this patient population are defined primarily by a subjective improvement in pain and daily function, rather than by structural healing status, at least in the early term. The limited sensitivity of entirely patient-reported instruments points to the necessity of objective functional assessments to capture the impact of RCR healing. Unlike the PROMs examined in this study, instruments such as the Constant-Murley^7^ or the University of California, Los Angeles score^1^ combine assessment of objective functional measures like shoulder strength or range of motion with patient-reported measures. PROMs that augment patient-assessment with functional measures have shown differences between RCR patients who heal and those who do not in previous studies.^30^ Notably, both the ASES score^28^ and PSS^22^ were developed to include an objective functional assessment component, but now only the patient-reported component is used. Our findings suggest that reintegrating objective functional assessments might be necessary to detect an effect of RCR healing, particularly in the early postoperative period.

Several limitations should be acknowledged. The study was conducted at a single tertiary center and included only fully reparable, 1–5 cm, isolated posterior superior cuff tears, all treated with a double-row repair. This narrows the generalizability of the findings to other patient populations who may be more responsive to healing, including those with higher activity levels, more severe tear types (for example, larger tears, or those involving the subscapularis tendon), or undergoing other surgical repair techniques. Additionally, only 5 shoulder PROMs were evaluated, leaving out other widely used instruments such as the Constant-Murley score, the Disability of the Arm, Shoulder, and Hand, and the Shoulder Pain and Disability Index,^15^ which may demonstrate different responsiveness to RCR and healing. Further, PROMs were assessed only in the 6 months to 2-year postoperative time-frame, limiting the ability to generalize these results to longer-term outcomes when patients with nonintact repairs may report lower PROMs compared to those with intact repairs.

## Conclusion

Several commonly utilized shoulder PROMs and their individual items—excluding SAL—demonstrated high responsiveness to RCR but not RCR healing during the first 2 postoperative years, with the majority of gains occurring within the first 6 months. In addition, 92% of patients achieved an acceptable symptom state by 1 year. Our findings emphasize the need for incorporating complementary outcome assessments, including objective functional measures such as shoulder strength or range of motion, in future studies to more fully characterize the relationship between RCR healing and postoperative recovery.

## Supplementary Material

1

Supplementary data to this article can be found online at https://doi.org/10.1016/j.jse.2026.01.010.

## Figures and Tables

**Table I –:** Demographics and rotator cuff tear characteristics (N = 113)

Variable	Statistics
Age, mean ± SD (yr)	58.5 ± 8.5
Sex, n (%)	
Female	49 (43.4)
Male	64 (56.6)
Race, n (%)	
White	98 (86.7)
Non-White	15 (13.3)
BMI, mean ± SD (kg/m^2^)	29.9 ± 5.9
Smoking, n (%)	
Yes	10 (8.8)
No	62 (54.9)
Quit	41 (36.3)
Employment, n (%)	
Employed	69 (61.1)
Not employed	7 (6.2)
Retired	37 (32.7)
AP tear length, mean ± SD (cm)	2.2 ± 0.86
ML tear length, mean ± SD (cm)	1.2 ± 0.52

*BMI*, body mass index; *SD*, standard deviation; *AP*, antero-posterior; *ML*, medio-lateral.

**Table II –:** PROMs before and after RCR (N=113)

PROMs	Mean ± SD				Floor effect (% at lowest possible score)				Ceiling effect (% at highest possible score)			
	Preoperative	6 mo	1 yr	2 yr	Preoperative	6 mo	1 yr	2 yr	Preoperative	6 mo	1 yr	2 yr
PSS-Total	42.2 ± 15.4	82.5 ± 14.8	90.2 ± 11.5	92.8 ± 10.9	0.0%	0.0%	0.0%	0.0%	0.0%	5.3%	12.4%	**30.1%**
PSS-Function	26.3 ± 10.0	48.9 ± 9.8	53.8 ± 7.9	55.7 ± 6.8	0.0%	0.0%	0.0%	0.0%	0.0%	8.9%	**21.2%**	**36.3%**
PSS-Pain	14.0 ± 5.7	26.0 ± 4.2	27.9 ± 3.0	28.1 ± 3.4	0.0%	0.0%	0.0%	0.0%	0.0%	**19.5%**	**38.1%**	**47.8%**
PSS-Satisfaction	1.8 ±2.1	7.5 ±2.5	8.6 ± 2.2	9.0 ± 2.0	**34.5%**	3.5%	3.5%	1.8%	1.8%	**21.2%**	**45.1%**	**59.3%**
ASES-Total	51.5 ± 17.5	85.4 ±13.9	91.8 ± 10.1	94.2 ± 9.6	0.0%	0.0%	0.0%	0.0%	0.0%	8.0%	**21.2%**	**36.3%**
ASES-Function	20.2 ± 8.0	39.1 ±8.7	43.8 ± 6.9	45.6 ± 6.4	0.0%	0.0%	0.0%	0.0%	0.0%	8.9%	**21.2%**	**37.2%**
ASES-Pain	31.2 ± 13.3	46.3 ± 7.1	48.0 ± 4.5	48.6 ± 4.0	3.5%	0.0%	0.0%	0.0%	7.1%	**65.5%**	**77.0%**	**83.2%**
SAL*	9.8 ± 4.8	10.1 ± 4.4	10.2 ± 4.0	9.9 ± 4.3	2.7%	2.7%	0.0%	4.4%	2.7%	0.0%	0.88%	0.0%
SANE	33.4 ± 20.1	77.8 ± 17.0	86.1 ± 15.5	89.6 ± 15.3	0.9%	0.0%	0.0%	0.9%	0.0%	6.2%	13.3%	**30.1%**
PROMIS-UE	31.7 ± 5.1	44.0 ± 8.1	48.4 ± 7.8	50.2 ± 7.9	0.9%	0.0%	0.0%	0.0%	0.0%	**23.0%**	**41.6%**	**54.0%**

*SANE*, Single Assessment Numeric Evaluation; *SAL*, Shoulder Activity Level; *PSS*, Penn Shoulder Score; *ASES*, American Shoulder and Elbow Surgeons; *PROM*, patient-reported outcome measure; Elbow Surgeons; *PROM*, patient-reported outcome measure; *PROMIS-UE*, Patient-Reported Outcome Measure Information System Upper Extremity; *SD*, standard deviation; *RCR*, rotator cuff repair.

Floor and ceiling effects >15% are considered unacceptable and are highlighted in bold.

* Postoperative improvements were statistically significant (*P* < .001) across all PROMs, except for SAL (*P* = .63, .42, and 0.84 at 6 mo, 1 y, and 2 y, respectively). Details of change scores, *P* values, and SRMs are provided in Supplementary Tables S6–S8.

**Table III –:** Number of healed vs. not healed patients for each healing definition, applied at each time point (N [%])

Healing status	Conventional definition	Stringent definition
At 6 mo		
Healed	91 (80.5%)	58 (51.3%)
Not healed	22 (19.5%)	18 (15.9%)
Not defined	-	37 (32.7%)
At 1 yr		
Healed	93 (82.3%)	47 (41.6%)
Not healed	20 (17.7%)	15 (13.3%)
Not defined	-	51 (45.1%)
At 2 yr		
Healed	94 (83.2%)	41 (36.3%)
Not healed	19 (16.8%)	15 (13.3%)
Not defined	-	57 (50.4%)

Conventional Definition: Healed: Sugaya scores 1–3; Not healed: Sugaya scores 4–5.

Stringent Definition: Healed: Sugaya scores 1–3 AND ≤10 mm tendon retraction; Not healed: Sugaya scores 4–5 AND ≥15 mm tendon retraction.

Imputation datasets accounting for missing data in Sugaya and tendon retraction were used.

**Table IV –:** Spearman correlations between PROMs and Sugaya classification (all categories) or tendon retraction (as a continuous variable) at 1 y

PROMs	Sugaya grade	Tendon retraction
PSS-Total	0.067	0.125
PSS-Function	0.096	0.115
PSS-Pain	0.016	0.089
PSS-Satisfaction	−0.003	0.174
ASES-Total	0.098	0.160
ASES-Function	0.109	0.133
ASES-Pain	−0.023	0.130
SAL	−0.002	0.064
SANE	0.027	0.098
PROMIS-UE	−0.025	0.114

*SANE*, Single Assessment Numeric Evaluation; *SAL*, Shoulder Activity Level; *PSS*, Penn Shoulder Score; *ASES*, American Shoulder and Elbow Surgeons; *PROM*, patient-reported outcome measure; *PROMIS-UE*, Patient-Reported Outcome Measure Information System Upper Extremity.

No correlations were significant at *P* < .05.

**Table V –:** Paired t-tests of PROM changes from preoperative to 1 year, for healed and not healed subgroups defined by conventional criteria at 1 year

PROMs	All (n = 113)			Healed (n = 93)			Not healed (n = 20)		
	Change (mean ± SD)	*P* value	SRM	Change (mean ± SD)	*P* value	SRM	Change (mean ± SD)	*P* value	SRM
PSS-Total	48.0 ± 16.9	**<.001**	2.84	47.2 ± 16.3	**<.001**	2.93	49.0 ± 19.7	**<.001**	2.49
PSS-Function	27.4 ± 10.8	**<.001**	2.53	27.3 ± 10.5	**<.001**	2.60	28.3 ± 12.6	**<.001**	2.25
PSS-Pain	13.8 ± 5.7	**<.001**	2.44	13.7 ± 5.7	**<.001**	2.42	14.3 ± 5.7	**<.001**	2.49
PSS-Satisfaction	6.7 ± 3.3	**<.001**	2.04	6.8 ± 3.4	**<.001**	2.00	6.4 ± 2.8	**<.001**	2.26
ASES-Total	40.3 ± 18.5	**<.001**	2.18	39.3 ± 17.7	**<.001**	2.22	44.8 ± 21.6	**<.001**	2.07
ASES-Function	23.5 ± 9.4	**<.001**	2.51	23.4 ± 9.2	**<.001**	2.55	24.1 ± 10.5	**<.001**	2.29
ASES-Pain	16.8 ± 13.4	**<.001**	1.25	15.9 ± 13.0	**<.001**	1.22	20.7 ± 14.6	**<.001**	1.42
SAL	0.3 ± 4.6	.420	0.08	0.0 ± 4.4	.960	0.00	2.1 ± 5.2	.087	0.40
SANE	52.7 ± 26.4	**<.001**	1.99	53.0 ± 25.7	**<.001**	2.07	51.0 ± 30.3	**<.001**	1.68
PROMIS-UE	16.7 ± 8.4	**<.001**	1.98	16.7 ± 8.1	**<.001**	2.06	16.4 ± 10.0	**<.001**	1.64

*SANE*, Single Assessment Numeric Evaluation; *SAL*, Shoulder Activity Level; *PSS*, Penn Shoulder Score; *ASES*, American Shoulder and Elbow Surgeons; *PROM*, patient-reported outcome measure; *PROMIS-UE*, Patient-Reported Outcome Measure Information System Upper Extremity; *SD*, standard deviation; *SRM*, standardized response mean.

*P* value based on paired t-test.

Patients were classified as healed vs. not healed at 1 y using observed data or mean of imputed values. Floor and ceiling effects >15% are considered unacceptable and are highlighted in bold.
