# Supplementary material for: Comparative responsiveness of shoulder patient-reported outcome measures (PROMs) to rotator cuff repair surgery and healing
Source: J Shoulder Elbow Surg. Author manuscript; Available in PMC 2026 Jul 16. (PMC13373755; doi:10.1016/j.jse.2026.01.010)
Supplement: 1 [file NIHMS2192031-supplement-1.docx]

**Supplementary material**

**Table S1.** Details of items within PSS, modified ASES*, SANE, PROMIS-UE, SAL and WORC score questionairres used in this study.

| **Score/Item** | **Description** | **Points** |
| --- | --- | --- |
| **PSS-Pain** | *Please select the number closest to your level of pain in your shoulder.* | **0-30** |
| PSS_pain_rest | Pain at rest with your arm by your side | 0-10 |
| PSS_pain_normal | Pain with normal activities (eating, dressing, bathing) | 0-10 |
| PSS_pain_strenuous | Pain with strenuous activities (reaching, lifting, pushing, pulling, throwing) | 0-10 |
| **PSS-Function** | *Please select the response that best describes the level of difficulty you might have performing each activity due to your shoulder.* | **0-60** |
| PSS f 01 | Reach the small of your back to tuck in your shirt with your hand | 0-3, each  3--No difficulty  2-- Some Difficulty  1-- Much difficulty 0-- Can’t do at all  X-- Did not do before injury |
| PSS f 02/ ASES f 03 | Wash the middle of your back/hook bra |  |
| PSS f 03/ ASES f 04 | Perform necessary toileting activities |  |
| PSS f 04 | Wash the back of opposite shoulder |  |
| PSS f 05/ ASES f 05 | Comb hair |  |
| PSS f 06 | Place hand behind head with elbow held straight out to the side |  |
| PSS f 07/ ASES f 01 | Dress self (including put on coat and pull shirt of overhead) |  |
| PSS f 08/ ASES f 02 | Sleep on affected side |  |
| PSS f 09 | Open a door with affected side |  |
| PSS f 10 | Carry a bag of groceries with affected arm |  |
| PSS f 11 | Carry a briefcase/small suitcase with affected arm |  |
| PSS f 12 | Place a soup can (1-2 lbs) on a shelf at shoulder level without bending elbow |  |
| PSS f 13 | Place a one-gallon container (8-10 lbs) on a shelf at shoulder level without bending elbow |  |
| PSS f 14/ ASES f 06 | Reach a shelf above your head without bending your elbow |  |
| PSS f 15 | Place a soup can (1-2 lbs) on a shelf overhead without bending your elbow |  |
| PSS f 16/ ASES f 07 | Place a one-gallon container (8-10 lbs) on a shelf overhead without bending your elbow |  |
| PSS f 17/ ASES f 10 | Perform usual sport/hobby |  |
| PSS f 18 | Perform household chores (cleaning, laundry, cooking) |  |
| PSS f 19/ ASES f 08 | Throw overhand/swim/overhead racquet sports |  |
| PSS f 20/ ASES f 09 | Work full-time at your regular job |  |
| **PSS-Satisfaction** | *Please select the number closest to your level of satisfaction with your shoulder.* | **0-10** |
| PSS satisfaction | How satisfied are you with the current level of function of your shoulder? | 0-10 |
| **ASES-Pain** | *Please select the number closest to your level of pain in your shoulder.* | **0-50** |
| ASES pain | How bad is your pain today? | 0-10 |
| **ASES-Function** | *Please select the response that best describes the level of difficulty you might have performing each activity due to your shoulder.* | **0-50*** |
| ASES f 01- ASES f 10 | 10 items shared with PSS Function (see above) | 0-3, each |
| **SANE** | How would you rate your shoulder today as a percentage of normal, with 100% being a normal shoulder? | **0-100** |
| **PROMIS-UE v1.2** | *Please answer the following questions to help us to understand how your shoulder is doing.* | **14.7-56.4** |
| promis_pfa29r1 | Are you able to pull heavy objects (10 pounds/ 5 kg) towards yourself?^¥^ | 1-5, each  5 - Without any difficulty  4 - With a little difficulty  3 - With some difficulty  2- With much difficulty  1- Unable to do |
| promis_pfa16r1 | Are you able to dress yourself, including tying shoelaces and buttoning your clothes? ^¥^ |  |
| promis_pfb22 | Are you able to hold a plate full of food? |  |
| promis_pfa38 | Are you able to dry your back with a towel? |  |
| promis_pfa17 | Are you able to reach into a high cupboard? |  |
| promis_pfa18 | Are you able to use a hammer to pound a nail? |  |
| promis_pfa28 | Are you able to open a can with a hand can opener? |  |
| promis_pfb36 | Are you able to put on a pullover sweater? |  |
| promis_pfb21 | Are you able to pick up coins from a table top? |  |
| promis_pfb33 | Are you able to remove something from your back pocket? |  |
| promis_pfb30 | Are you able to open a new milk carton? |  |
| promis_pfa48 | Are you able to peel fruit? |  |
| promis_pfa44 | Are you able to put on a shirt or blouse? |  |
| promis_pfa20 | Are you able to cut your food using eating utensils? ^#^ |  |
| promis_pfa35 | Are you able to open and close a zipper? ^#^ |  |
| promis_pfa54 | Are you able to button your shirt? ^#^ |  |
| **Shoulder Activity Level (SAL)** | *Please answer the following questions regarding your typical level of activity over the last year. Answer these for how often you perform these activities, not the level of difficulty performing them.* | **0-20 points** |
| pro_marx_1 | Carrying objects 8 pounds or heavier by hand (such as a bag of groceries) | 0-4, each  0-Never or less than once a month;  1-Once a Month;  2- Once a week;  3- More than once a week;  4- Daily. |
| pro_marx_2 | Handling objects overhead |  |
| pro_marx_3 | Weightlifting or weight training with arms |  |
| pro_marx_4 | Swinging motion (as in hitting a tennis ball, golf ball, baseball, or similar object) |  |
| pro_marx_5 | Lifting objects 25 pounds or heavier (such as 3 gallons of water) NOT INCLUDING WEIGHTLIFTING |  |
| **Western Ontario Rotator Cuff Index (WORC)** | *Please answer the following questions for your shoulder.* | **Not scored** |
| Work_11 | How much difficulty do you experience in daily activities about the house or yard? | 0 - do not experience that symptom;  100 - symptom is extreme. |
| Work_12 | How much difficulty do you experience working above your shoulder? |  |
| Work_13 | How much do you use your uninvolved arm to compensate for your injured one? |  |
| Work_14 | How much difficulty do you experience lifting heavy objects at or below shoulder level? |  |
| Lifestyle_15 | How much difficulty do you have sleeping because of your shoulder? |  |
| Lifestyle_16 | How much difficulty have you experienced with styling your hair because of your shoulder? |  |
| Lifestyle_18 | How much difficulty do you have dressing or undressing? |  |

^¥^Always or ^#^never asked in our cohort

^#^ Modified ASES-Function items: we used the ten PSS-Function items, which are reworded versions of the original ASES-Function items and include an additional response option: “X-- Did not do before injury”.

Modified ASES-Function score: [(Sum of responses to ASES-Function items, excluding those marked “X’) / (Number of ASES-Function items without an ‘X’ response)] x 50/3

This modified calculation differs from the original ASES-Function scoring method which simply sums the item responses and multiplies by 5/3.

**Table S2.** PROM items before and after RCR (N=113)

|  | **Pre-op** | | **6-month** | | **1-year** | | **2-year** | |
| --- | --- | --- | --- | --- | --- | --- | --- | --- |
| **Pain Items** | **n** | **Mean ±SD** | **n** | **Mean ±SD** | **n** | **Mean ±SD** | **n** | **Mean ±SD** |
| PSS pain at rest | 113 | 2.7 ±2.4 | 113 | 0.36 ±0.78 | 113 | 0.17 ±0.52 | 113 | 0.25 ±0.82 |
| PSS pain normal activities | 113 | 5.1 ±2.5 | 113 | 1.1 ±1.6 | 113 | 0.52 ±1.1 | 113 | 0.43 ±1.2 |
| PSS pain strenuous activities | 113 | 8.1 ±1.8 | 113 | 2.5 ±2.3 | 113 | 1.4 ±1.7 | 113 | 1.2 ±1.8 |
| ASES pain today | 113 | 3.8 ±2.7 | 113 | 0.74 ±1.4 | 113 | 0.40 ±0.89 | 113 | 0.28 ±0.81 |
| **Function Items** |  |  |  |  |  |  |  |  |
| PSS f 01 | 113 | 1.5 ±0.79 | 113 | 2.3 ±0.75 | 113 | 2.7 ±0.56 | 113 | 2.8 ±0.53 |
| PSS f 02/ ASES f 03 | 112 | 0.96 ±0.82 | 112 | 1.8 ±0.98 | 109 | 2.4 ±0.78 | 111 | 2.5 ±0.68 |
| PSS f 03/ ASES f 04 | 112 | 2.1 ±0.86 | 113 | 2.8 ±0.38 | 112 | 2.9 ±0.26 | 112 | 2.9 ±0.29 |
| PSS f 04 | 113 | 1.1 ±0.95 | 113 | 2.2 ±0.80 | 112 | 2.6 ±0.62 | 113 | 2.8 ±0.45 |
| PSS f 05/ ASES f 05 | 110 | 1.7 ±0.86 | 113 | 2.8 ±0.47 | 112 | 2.9 ±0.34 | 112 | 2.9 ±0.29 |
| PSS f 06 | 113 | 1.4 ±0.90 | 113 | 2.5 ±0.76 | 113 | 2.7 ±0.58 | 113 | 2.8 ±0.50 |
| PSS f 07/ ASES f 01 | 113 | 1.7 ±0.59 | 113 | 2.7 ±0.47 | 113 | 2.8 ±0.39 | 113 | 2.9 ±0.36 |
| PSS f 08/ ASES f 02 | 112 | 1.05 ±0.79 | 113 | 2.3 ±0.70 | 112 | 2.6 ±0.64 | 110 | 2.7 ±0.51 |
| PSS f 09 | 113 | 1.9 ±0.75 | 113 | 2.8 ±0.46 | 113 | 2.9 ±0.32 | 113 | 2.9 ±0.26 |
| PSS f 10 | 113 | 1.6 ±0.78 | 113 | 2.7 ±0.52 | 113 | 2.8 ±0.48 | 113 | 2.9 ±0.29 |
| PSS f 11 | 113 | 1.8 ±0.84 | 112 | 2.8 ±0.49 | 112 | 2.8 ±0.48 | 113 | 2.9 ±0.30 |
| PSS f 12 | 113 | 1.4 ±0.87 | 113 | 2.7 ±0.51 | 113 | 2.9 ±0.31 | 113 | 2.9 ±0.37 |
| PSS f 13 | 113 | 0.73 ±0.79 | 112 | 2.1 ±0.90 | 113 | 2.5 ±0.76 | 113 | 2.6 ±0.68 |
| PSS f 14/ ASES f 06 | 113 | 0.99 ±0.82 | 113 | 2.4 ±0.79 | 113 | 2.8 ±0.55 | 113 | 2.8 ±0.43 |
| PSS f 15 | 113 | 1.01 ±0.86 | 113 | 2.5 ±0.81 | 113 | 2.7 ±0.60 | 113 | 2.8 ±0.47 |
| PSS f 16/ ASES f 07 | 113 | 0.50 ±0.66 | 112 | 1.8 ±1.00 | 112 | 2.2 ±0.85 | 113 | 2.4 ±0.77 |
| PSS f 17/ ASES f 10 | 107 | 0.97 ±0.82 | 112 | 2.2 ±0.86 | 109 | 2.6 ±0.60 | 108 | 2.7 ±0.57 |
| PSS f 18 | 113 | 1.8 ±0.67 | 113 | 2.8 ±0.42 | 113 | 2.8 ±0.41 | 113 | 2.9 ±0.30 |
| PSS f 19/ ASES f 08 | 105 | 0.41 ±0.57 | 89 | 1.9 ±0.85 | 98 | 2.3 ±0.78 | 98 | 2.6 ±0.69 |
| PSS f 20/ ASES f 09 | 101 | 1.8 ±0.86 | 92 | 2.7 ±0.66 | 95 | 2.8 ±0.48 | 92 | 2.9 ±0.42 |
| SAL 1 | 113 | 2.9 ±1.06 | 113 | 2.9 ±1.02 | 113 | 3.0 ±0.93 | 113 | 2.9 ±1.08 |
| SAL 2 | 113 | 3.0 ±1.2 | 113 | 2.9 ±1.2 | 113 | 3.0 ±1.06 | 113 | 3.0 ±1.1 |
| SAL 3 | 113 | 1.2 ±1.5 | 113 | 1.6 ±1.5 | 113 | 1.5 ±1.5 | 113 | 1.2 ±1.4 |
| SAL 4 | 113 | 1.00 ±1.3 | 113 | 1.01 ±1.3 | 113 | 0.96 ±1.2 | 113 | 1.05 ±1.3 |
| SAL 5 | 113 | 1.8 ±1.4 | 113 | 1.7 ±1.4 | 113 | 1.8 ±1.3 | 113 | 1.8 ±1.3 |
| WORC 11 | 113 | 63.0 ±21.5 | 113 | 22.9 ±22.8 | 113 | 13.9 ±21.2 | 113 | 12.0 ±23.5 |
| WORC 12 | 113 | 81.7 ±20.0 | 113 | 36.6 ±29.3 | 113 | 23.8 ±27.6 | 113 | 20.7 ±27.9 |
| WORC 13 | 113 | 74.6 ±19.4 | 113 | 34.7 ±30.9 | 113 | 20.0 ±27.0 | 113 | 15.8 ±25.3 |
| WORC 14 | 113 | 66.2 ±27.4 | 113 | 30.0 ±27.9 | 113 | 18.3 ±24.5 | 113 | 18.0 ±27.7 |
| WORC 15 | 113 | 69.5 ±25.5 | 113 | 18.6 ±24.1 | 113 | 10.9 ±19.1 | 113 | 8.5 ±18.3 |
| WORC 16 | 113 | 60.9 ±28.2 | 113 | 13.4 ±22.7 | 113 | 6.8 ±16.2 | 113 | 4.5 ±12.0 |
| WORC 18 | 113 | 54.6 ±23.1 | 113 | 9.0 ±13.3 | 113 | 5.1 ±13.5 | 113 | 3.6 ±10.3 |
| promis_pfa29r1 | 113 | 2.9 ±1.05 | 113 | 4.3 ±0.98 | 113 | 4.6 ±0.74 | 113 | 4.7 ±0.83 |
| promis_pfa16r1 | 113 | 3.6 ±0.96 | 113 | 4.7 ±0.55 | 113 | 4.8 ±0.38 | 113 | 4.8 ±0.47 |
| promis_pfb22 | 112 | 4.0 ±1.04 | 108 | 4.9 ±0.40 | 113 | 4.9 ±0.28 | 113 | 4.9 ±0.39 |
| promis_pfa38 | 55 | 3.6 ±1.00 | 106 | 4.4 ±0.88 | 111 | 4.7 ±0.60 | 110 | 4.8 ±0.57 |
| promis_pfa17 | 18 | 3.2 ±1.04 | 96 | 4.3 ±0.82 | 101 | 4.6 ±0.62 | 104 | 4.7 ±0.65 |
| promis_pfa18 | 18 | 4.3 ±0.89 | 91 | 4.7 ±0.69 | 100 | 4.9 ±0.38 | 103 | 4.9 ±0.29 |
| promis_pfa44 | 80 | 3.2 ±0.88 | 40 | 4.6 ±0.59 | 26 | 4.8 ±0.40 | 21 | 4.7 ±0.64 |
| promis_pfa28 | 4 | 5 ±0 | 70 | 4.9 ±0.35 | 91 | 4.9 ±0.27 | 97 | 4.9 ±0.5 |
| promis_pfb36 | 4 | 3.8 ±0.5 | 70 | 4.8 ±0.38 | 90 | 5 ±0.18 | 96 | 4.9 ±0.3 |
| promis_pfb21 | 1 | 5 | 59 | 5 ±0.13 | 87 | 5 ±0.18 | 92 | 5 ±0.15 |
| promis_pfb33 | 2 | 5 ±0 | 58 | 4.9 ±0.38 | 87 | 5 ±0.21 | 91 | 5 ±0.23 |
| promis_pfb30 | 1 | 5 | 54 | 5 ±0.19 | 85 | 5 ±0.11 | 91 | 5 ±0 |
| promis_pfa48 | 0 | - | 39 | 5 ±0 | 67 | 5 ±0 | 81 | 5 ±0 |

**Table S3.** Sugaya Classification (N (column %)) and Tendon Retraction (Mean ±SD) data of patients at 6-month, 1-year and 2-year following RCR

| **Healing measures** | | **6-month (N=113)** | **1-year (N=113)** | **2-year (N=111)** |
| --- | --- | --- | --- | --- |
| Sugaya | 1 | - | - | 1 (0.90) |
|  | 1.5 | 3 (2.7) | - | 5 (4.5) |
|  | 2 | 59 (52.2) | 70 (61.9) | 65 (58.6) |
|  | 3 | 29 (25.7) | 23 (20.4) | 21 (18.9) |
|  | 4 | 10 (8.8) | 8 (7.1) | 4 (3.6) |
|  | 4.5 | 1 (0.88) | 2 (1.8) | 4 (3.6) |
|  | 5 | 11 (9.7) | 10 (8.8) | 11 (9.9) |
| Tendon retraction, mm | | 11.8 ±7.0 (n=85) | 12.5 ±7.4 (n=80) | 12.9 ±7.1 (n=75) |

**Table S4.** Spearman correlations between PROMs and Sugaya classification (all categories) or tendon retraction (as a continuous variable) at 6-month and 2-year

|  | **6-month** | | **2-year** | |
| --- | --- | --- | --- | --- |
|  | **Sugaya Grade** | **Tendon Retraction** | **Sugaya Grade** | **Tendon Retraction** |
| **PSS-Total** | 0.146 | 0.219* | 0.200* | 0.106 |
| PSS-Function | 0.169 | 0.236* | 0.172 | 0.129 |
| PSS-Pain | 0.160 | 0.115 | 0.110 | 0.027 |
| PSS-Satisfaction | 0.085 | 0.207* | 0.244* | 0.142 |
| **ASES-Total** | 0.145 | 0.174 | 0.180 | 0.127 |
| ASES-Function | 0.178 | 0.223* | 0.186 | 0.126 |
| ASES-Pain | 0.014 | 0.030 | 0.062 | 0.049 |
| **SAL** | -0.043 | -0.022 | 0.023 | -0.009 |
| **SANE** | 0.150 | 0.184 | 0.088 | 0.120 |
| **PROMIS-UE** | 0.087 | 0.218* | 0.065 | 0.018 |

*Significant at p<0.05 but in the opposite direction as hypothesized; better PROMs associated with worse healing

**Table S5.** Spearman correlations between PROM items and Sugaya classification (all categories) or tendon retraction (as a continuous variable) at 6-month, 1-year, and 2-year

|  | **At 6-month** | | **At 1-year** | | **At 2-year** | |
| --- | --- | --- | --- | --- | --- | --- |
|  | **Sugaya Grade** | **Tendon Retraction** | **Sugaya Grade** | **Tendon Retraction** | **Sugaya Grade** | **Tendon Retraction** |
| **Pain Items** |  |  |  |  |  |  |
| PSS pain at rest | -0.096 | -0.139 | -0.166 | -0.208* | -0.102 | -0.042 |
| PSS pain normal activities | -0.166 | -0.044 | 0.055 | -0.009 | -0.083 | -0.001 |
| PSS pain strenuous activities | -0.142 | -0.112 | -0.011 | -0.051 | -0.088 | -0.034 |
| ASES pain today | -0.014 | -0.030 | 0.023 | -0.130 | -0.062 | -0.049 |
| **Function Items** |  |  |  |  |  |  |
| PSS f 01 | 0.195* | 0.259* | -0.074 | 0.102 | 0.120 | 0.145 |
| PSS f 02/ ASES f 03 | 0.196* | 0.226* | 0.060 | 0.161 | 0.205* | 0.120 |
| PSS f 03/ ASES f 04 | 0.127 | 0.211* | 0.072 | 0.094 | 0.084 | 0.092 |
| PSS f 04 | 0.189* | 0.252* | 0.149 | 0.227* | 0.163 | 0.077 |
| PSS f 05/ ASES f 05 | 0.049 | 0.201* | 0.006 | 0.007 | -0.028 | -0.152 |
| PSS f 06 | 0.103 | 0.186 | -0.000 | 0.021 | 0.110 | 0.045 |
| PSS f 07/ ASES f 01 | 0.137 | 0.169 | 0.131 | 0.225* | 0.165 | 0.155 |
| PSS f 08/ ASES f 02 | 0.265* | 0.068 | 0.134 | 0.158 | 0.077 | 0.041 |
| PSS f 09 | 0.094 | 0.111 | 0.028 | -0.015 | 0.032 | -0.152 |
| PSS f 10 | 0.108 | 0.179 | -0.068 | -0.014 | 0.119 | 0.025 |
| PSS f 11 | 0.062 | 0.069 | 0.040 | -0.042 | 0.172 | 0.023 |
| PSS f 12 | 0.119 | 0.062 | -0.006 | 0.003 | 0.036 | -0.040 |
| PSS f 13 | 0.023 | 0.106 | -0.046 | -0.065 | 0.028 | -0.040 |
| PSS f 14/ ASES f 06 | 0.088 | 0.147 | -0.094 | 0.029 | 0.003 | -0.018 |
| PSS f 15 | 0.094 | 0.082 | 0.029 | 0.056 | 0.004 | -0.041 |
| PSS f 16/ ASES f 07 | 0.022 | 0.093 | -0.035 | -0.028 | 0.085 | -0.018 |
| PSS f 17/ ASES f 10 | 0.171 | 0.262* | 0.195* | 0.271* | 0.287* | 0.146 |
| PSS f 18 | 0.066 | 0.125 | 0.003 | -0.038 | 0.120 | 0.018 |
| PSS f 19/ ASES f 08 | 0.149 | 0.243* | 0.013 | 0.067 | 0.101 | 0.131 |
| PSS f 20/ ASES f 09 | 0.026 | 0.029 | 0.083 | -0.100 | 0.154 | 0.006 |
| SAL 1 | -0.058 | 0.073 | 0.022 | 0.029 | 0.005 | 0.045 |
| SAL 2 | -0.059 | 0.064 | -0.030 | 0.093 | -0.146 | 0.002 |
| SAL 3 | 0.026 | -0.128 | 0.065 | 0.039 | 0.098 | -0.026 |
| SAL 4 | -0.048 | -0.059 | 0.070 | 0.117 | 0.112 | 0.000 |
| SAL 5 | 0.019 | 0.062 | -0.017 | 0.062 | 0.037 | 0.014 |
| WORC 11 | -0.141 | -0.105 | -0.118 | -0.041 | -0.079 | 0.009 |
| WORC 12 | -0.180 | -0.175 | -0.078 | -0.098 | -0.084 | 0.033 |
| WORC 13 | -0.150 | -0.205* | -0.104 | -0.073 | -0.047 | -0.053 |
| WORC 14 | -0.105 | -0.203* | -0.056 | 0.014 | -0.017 | 0.023 |
| WORC 15 | -0.220* | -0.203* | -0.104 | -0.124 | -0.085 | -0.056 |
| WORC 16 | -0.086 | -0.209* | -0.015 | -0.054 | -0.117 | -0.074 |
| WORC 18 | -0.135 | -0.296* | -0.193* | -0.178 | -0.119 | -0.160 |
| promis_pfa29r1 | 0.014 | 0.056 | -0.144 | -0.056 | 0.123 | -0.038 |
| promis_pfa16r1 | 0.116 | 0.155 | 0.092 | 0.122 | 0.041 | -0.039 |
| promis_pfb22 | -0.001 | 0.002 | 0.026 | -0.120 | 0.108 | -0.041 |

*Significant at p<0.05.

Note that correlations, though weak or very small, were in the opposite direction to what was expected.

**Table S6.** Paired t-tests of PROM total scores and item-level changes from pre-op to 6-month, for healed and not healed subgroups defined by *conventional* criteria at 6-month

|  | **All (n=113)** | | | **Healed (n=91)** | | | **Not Healed (n=22)** | | |
| --- | --- | --- | --- | --- | --- | --- | --- | --- | --- |
|  | **Change**  **Mean (SD)** | **p-value** | **SRM** | **Change**  **Mean (SD)** | **p-value** | **SRM** | **Change**  **Mean (SD)** | **p-value** | **SRM** |
| **PSS-Total** | 40.30 (16.43) | ***<0.001*** | 2.45 | 39.34 (15.78) | ***<0.001*** | **2.49** | 44.26 (18.74) | ***<0.001*** | **2.36** |
| PSS-Function | 22.62 (10.84) | ***<0.001*** | 2.09 | 21.85 (10.44) | ***<0.001*** | 2.09 | 25.80 (12.08) | ***<0.001*** | 2.14 |
| PSS f 01 | 0.86 (0.89) | ***<0.001*** | 0.97 | 0.79 (0.85) | ***<0.001*** | 0.93 | 1.14 (0.99) | ***<0.001*** | 1.15 |
| PSS f 02/ ASES f 03 | 0.89 (1.02) | ***<0.001*** | 0.87 | 0.82 (0.98) | ***<0.001*** | 0.83 | 1.18 (1.14) | ***<0.001*** | 1.04 |
| PSS f 03/ ASES f 04 | 0.78 (0.80) | ***<0.001*** | 0.97 | 0.78 (0.83) | ***<0.001*** | 0.94 | 0.77 (0.69) | ***<0.001*** | 1.13 |
| PSS f 04 | 1.08 (1.02) | ***<0.001*** | 1.06 | 1.03 (1.06) | ***<0.001*** | 0.98 | 1.27 (0.83) | ***<0.001*** | 1.54 |
| PSS f 05/ ASES f 05 | 1.10 (0.89) | ***<0.001*** | 1.24 | 1.09 (0.88) | ***<0.001*** | 1.24 | 1.14 (0.94) | ***<0.001*** | 1.21 |
| PSS f 06 | 1.07 (1.11) | ***<0.001*** | 0.97 | 1.03 (1.07) | ***<0.001*** | 0.97 | 1.23 (1.27) | ***<0.001*** | 0.97 |
| PSS f 07/ ASES f 01 | 0.96 (0.71) | ***<0.001*** | 1.37 | 0.92 (0.67) | ***<0.001*** | 1.38 | 1.14 (0.83) | ***<0.001*** | 1.36 |
| PSS f 08/ ASES f 02 | 1.26 (1.06) | ***<0.001*** | 1.18 | 1.26 (1.07) | ***<0.001*** | 1.18 | 1.27 (1.08) | ***<0.001*** | 1.18 |
| PSS f 09 | 0.92 (0.76) | ***<0.001*** | 1.21 | 0.91 (0.77) | ***<0.001*** | 1.19 | 0.95 (0.72) | ***<0.001*** | 1.32 |
| PSS f 10 | 1.11 (0.79) | ***<0.001*** | 1.39 | 1.03 (0.80) | ***<0.001*** | 1.30 | 1.41 (0.73) | ***<0.001*** | 1.92 |
| PSS f 11 | 0.96 (0.80) | ***<0.001*** | 1.20 | 0.88 (0.79) | ***<0.001*** | 1.11 | 1.27 (0.77) | ***<0.001*** | 1.66 |
| PSS f 12 | 1.36 (0.91) | ***<0.001*** | 1.50 | 1.35 (0.91) | ***<0.001*** | 1.48 | 1.41 (0.91) | ***<0.001*** | 1.55 |
| PSS f 13 | 1.40 (0.99) | ***<0.001*** | 1.42 | 1.37 (1.02) | ***<0.001*** | 1.34 | 1.55 (0.86) | ***<0.001*** | 1.80 |
| PSS f 14/ ASES f 06 | 1.41 (0.96) | ***<0.001*** | 1.47 | 1.36 (0.95) | ***<0.001*** | 1.44 | 1.59 (1.01) | ***<0.001*** | 1.58 |
| PSS f 15 | 1.49 (1.02) | ***<0.001*** | 1.46 | 1.46 (1.01) | ***<0.001*** | 1.44 | 1.59 (1.05) | ***<0.001*** | 1.51 |
| PSS f 16/ ASES f 07 | 1.33 (0.92) | ***<0.001*** | 1.44 | 1.31 (0.97) | ***<0.001*** | 1.36 | 1.41 (0.73) | ***<0.001*** | 1.92 |
| PSS f 17/ ASES f 10 | 1.19 (1.00) | ***<0.001*** | 1.19 | 1.08 (1.01) | ***<0.001*** | 1.07 | 1.62 (0.86) | ***<0.001*** | 1.87 |
| PSS f 18 | 1.01 (0.69) | ***<0.001*** | 1.47 | 0.99 (0.66) | ***<0.001*** | 1.50 | 1.09 (0.81) | ***<0.001*** | 1.34 |
| PSS f 19/ ASES f 08 | 1.45 (0.83) | ***<0.001*** | 1.74 | 1.44 (0.80) | ***<0.001*** | 1.80 | 1.50 (1.02) | ***<0.001*** | 1.47 |
| PSS f 20/ ASES f 09 | 0.86 (0.82) | ***<0.001*** | 1.04 | 0.84 (0.82) | ***<0.001*** | 1.02 | 0.94 (0.87) | ***<0.001*** | 1.08 |
| PSS-Pain | 12.00 (5.33) | ***<0.001*** | 2.25 | 11.76 (5.17) | ***<0.001*** | 2.27 | 13.00 (5.95) | ***<0.001*** | 2.18 |
| PSS pain at rest | -2.38 (2.34) | ***<0.001*** | -1.02 | -2.20 (2.13) | ***<0.001*** | -1.03 | -3.14 (3.03) | ***<0.001*** | -1.04 |
| PSS pain normal activities | -4.01 (2.33) | ***<0.001*** | -1.72 | -3.88 (2.30) | ***<0.001*** | -1.69 | -4.55 (2.42) | ***<0.001*** | -1.87 |
| PSS pain strenuous activities | -5.61 (2.21) | ***<0.001*** | -2.54 | -5.68 (2.08) | ***<0.001*** | -2.74 | -5.32 (2.73) | ***<0.001*** | -1.95 |
| PSS-Satisfaction | 5.68 (2.98) | ***<0.001*** | 1.90 | 5.74 (2.87) | ***<0.001*** | 2.00 | 5.45 (3.47) | ***<0.001*** | 1.57 |
| **ASES-Total** | 33.93 (19.15) | ***<0.001*** | 1.77 | 32.82 (18.03) | ***<0.001*** | **1.82** | 38.49 (23.14) | ***<0.001*** | **1.66** |
| ASES-Function | 18.88 (9.52) | ***<0.001*** | 1.98 | 18.26 (9.14) | ***<0.001*** | 2.00 | 21.44 (10.81) | ***<0.001*** | 1.98 |
| ASES-Pain | 15.04 (13.37) | ***<0.001*** | 1.13 | 14.56 (13.12) | ***<0.001*** | 1.11 | 17.05 (14.53) | ***<0.001*** | 1.17 |
| ASES pain today | -3.01 (2.67) | ***<0.001*** | -1.13 | -2.91 (2.62) | ***<0.001*** | -1.11 | -3.41 (2.91) | ***<0.001*** | -1.17 |
| **SAL** | 0.22 (4.89) | 0.63 | 0.05 | -0.23 (4.55) | 0.63 | -**0.05** | 2.09 (5.85) | 0.11 | **0.36** |
| SAL 1 | -0.04 (1.26) | 0.71 | -0.04 | -0.13 (1.21) | 0.30 | -0.11 | 0.32 (1.43) | 0.31 | 0.22 |
| SAL 2 | -0.08 (1.53) | 0.58 | -0.05 | -0.23 (1.36) | 0.11 | -0.17 | 0.55 (2.02) | 0.22 | 0.27 |
| SAL 3 | 0.43 (1.43) | ***0.002*** | 0.30 | 0.35 (1.44) | ***0.022*** | 0.24 | 0.77 (1.34) | ***0.013*** | 0.58 |
| SAL 4 | 0.01 (1.25) | 0.94 | 0.01 | -0.03 (1.17) | 0.79 | -0.03 | 0.18 (1.56) | 0.59 | 0.12 |
| SAL 5 | -0.10 (1.51) | 0.50 | -0.06 | -0.19 (1.34) | 0.19 | -0.14 | 0.27 (2.07) | 0.54 | 0.13 |
| **SANE** | 44.41 (25.23) | ***<0.001*** | 1.76 | 42.77 (25.13) | ***<0.001*** | **1.70** | 51.18 (25.07) | ***<0.001*** | **2.04** |
| **WORC** | - | - | - | - | ***-*** | - | - | ***-*** | **-** |
| WORC 11 | -40.03 (29.81) | ***<0.001*** | -1.34 | -38.15 (27.23) | ***<0.001*** | -1.40 | -47.77 (38.53) | ***<0.001*** | -1.24 |
| WORC 12 | -45.11 (33.73) | ***<0.001*** | -1.34 | -43.88 (31.51) | ***<0.001*** | -1.39 | -50.18 (42.19) | ***<0.001*** | -1.19 |
| WORC 13 | -39.89 (32.11) | ***<0.001*** | -1.24 | -37.91 (30.51) | ***<0.001*** | -1.24 | -48.05 (37.70) | ***<0.001*** | -1.27 |
| WORC 14 | -36.19 (34.18) | ***<0.001*** | -1.06 | -35.40 (31.33) | ***<0.001*** | -1.13 | -39.50 (44.83) | ***<0.001*** | -0.88 |
| WORC 15 | -50.83 (31.91) | ***<0.001*** | -1.59 | -51.51 (28.76) | ***<0.001*** | -1.79 | -48.05 (43.34) | ***<0.001*** | -1.11 |
| WORC 16 | -47.44 (31.81) | ***<0.001*** | -1.49 | -48.84 (27.17) | ***<0.001*** | -1.80 | -41.68 (46.79) | ***<0.001*** | -0.89 |
| WORC 18 | -45.55 (23.69) | ***<0.001*** | -1.92 | -45.69 (21.50) | ***<0.001*** | -2.13 | -44.95 (31.82) | ***<0.001*** | -1.41 |
| **PROMIS-UE** | 12.28 (7.95) | ***<0.001*** | 1.54 | 12.06 (7.65) | ***<0.001*** | **1.58** | 13.22 (9.24) | ***<0.001*** | **1.43** |
| promis_pfa29r1 | 1.37 (1.14) | ***<0.001*** | 1.20 | 1.36 (1.08) | ***<0.001*** | 1.26 | 1.41 (1.40) | ***<0.001*** | 1.00 |
| promis_pfa16r1 | 1.14 (0.93) | ***<0.001*** | 1.22 | 1.13 (0.93) | ***<0.001*** | 1.21 | 1.18 (0.96) | ***<0.001*** | 1.23 |
| promis_pfb22 | 0.91 (0.96) | ***<0.001*** | 0.95 | 0.84 (0.91) | ***<0.001*** | 0.92 | 1.20 (1.11) | ***<0.001*** | 1.09 |

*p-value based on paired t-test; SRM = standardized response mean.

Note that not all PROM items had n=113, as certain PSS function items were not applicable to some patients, and some PROMIS-UE items were not administered by the CAT to some patients, as detailed in **Table S1**.

Patients were classified as healed vs not healed at 6-month using observed data or mean of imputed values.

**Table S7.** Paired t-tests of PROM total scores and item level changes from pre-op to 1-year, for healed and not healed subgroups defined by *conventional* criteria at 1-year.

|  | **All (n=113)** | | | **Healed (n=93)** | | | | **Not Healed (n=20)** | | |
| --- | --- | --- | --- | --- | --- | --- | --- | --- | --- | --- |
|  | **Change Mean (SD)** | **p-value** | **SRM** | **Change Mean (SD)** | **p-value** | **SRM** | **Change Mean (SD)** | | **p-value** | **SRM** |
| **PSS-Total** | 48.02 (16.88) | ***<0.001*** | 2.84 | 47.82 (16.33) | ***<0.001*** | 2.93 | 48.96 (19.70) | | ***<0.001*** | 2.49 |
| PSS-Function | 27.44 (10.83) | ***<0.001*** | 2.53 | 27.27 (10.49) | ***<0.001*** | 2.60 | 28.26 (12.58) | | ***<0.001*** | 2.25 |
| PSS f 01 | 1.21 (0.88) | ***<0.001*** | 1.38 | 1.24 (0.89) | ***<0.001*** | 1.39 | 1.10 (0.85) | | ***<0.001*** | 1.29 |
| PSS f 02/ ASES f 03 | 1.42 (0.93) | ***<0.001*** | 1.53 | 1.42 (0.92) | ***<0.001*** | 1.54 | 1.39 (0.98) | | ***<0.001*** | 1.42 |
| PSS f 03/ ASES f 04 | 0.87 (0.81) | ***<0.001*** | 1.08 | 0.88 (0.81) | ***<0.001*** | 1.09 | 0.84 (0.83) | | ***<0.001*** | 1.01 |
| PSS f 04 | 1.49 (0.92) | ***<0.001*** | 1.62 | 1.48 (0.95) | ***<0.001*** | 1.55 | 1.55 (0.76) | | ***<0.001*** | 2.04 |
| PSS f 05/ ASES f 05 | 1.23 (0.89) | ***<0.001*** | 1.38 | 1.22 (0.87) | ***<0.001*** | 1.40 | 1.26 (0.99) | | ***<0.001*** | 1.27 |
| PSS f 06 | 1.28 (1.00) | ***<0.001*** | 1.29 | 1.22 (0.98) | ***<0.001*** | 1.24 | 1.60 (1.05) | | ***<0.001*** | 1.53 |
| PSS f 07/ ASES f 01 | 1.13 (0.65) | ***<0.001*** | 1.75 | 1.11 (0.58) | ***<0.001*** | 1.91 | 1.25 (0.91) | | ***<0.001*** | 1.37 |
| PSS f 08/ ASES f 02 | 1.55 (0.93) | ***<0.001*** | 1.66 | 1.55 (0.95) | ***<0.001*** | 1.63 | 1.53 (0.84) | | ***<0.001*** | 1.81 |
| PSS f 09 | 1.05 (0.74) | ***<0.001*** | 1.42 | 1.06 (0.73) | ***<0.001*** | 1.45 | 1.00 (0.79) | | ***<0.001*** | 1.26 |
| PSS f 10 | 1.17 (0.83) | ***<0.001*** | 1.40 | 1.15 (0.82) | ***<0.001*** | 1.40 | 1.25 (0.91) | | ***<0.001*** | 1.37 |
| PSS f 11 | 1.03 (0.85) | ***<0.001*** | 1.20 | 0.98 (0.86) | ***<0.001*** | 1.13 | 1.25 (0.79) | | ***<0.001*** | 1.59 |
| PSS f 12 | 1.51 (0.87) | ***<0.001*** | 1.74 | 1.49 (0.84) | ***<0.001*** | 1.77 | 1.60 (0.99) | | ***<0.001*** | 1.61 |
| PSS f 13 | 1.74 (1.02) | ***<0.001*** | 1.70 | 1.76 (1.03) | ***<0.001*** | 1.72 | 1.65 (1.04) | | ***<0.001*** | 1.59 |
| PSS f 14/ ASES f 06 | 1.79 (0.90) | ***<0.001*** | 1.98 | 1.76 (0.91) | ***<0.001*** | 1.93 | 1.90 (0.85) | | ***<0.001*** | 2.23 |
| PSS f 15 | 1.73 (0.95) | ***<0.001*** | 1.82 | 1.73 (0.93) | ***<0.001*** | 1.85 | 1.70 (1.03) | | ***<0.001*** | 1.65 |
| PSS f 16/ ASES f 07 | 1.68 (0.93) | ***<0.001*** | 1.80 | 1.70 (0.96) | ***<0.001*** | 1.77 | 1.60 (0.82) | | ***<0.001*** | 1.95 |
| PSS f 17/ ASES f 10 | 1.61 (0.95) | ***<0.001*** | 1.70 | 1.59 (0.95) | ***<0.001*** | 1.68 | 1.68 (0.95) | | ***<0.001*** | 1.78 |
| PSS f 18 | 1.09 (0.68) | ***<0.001*** | 1.61 | 1.09 (0.65) | ***<0.001*** | 1.66 | 1.10 (0.79) | | ***<0.001*** | 1.40 |
| PSS f 19/ ASES f 08 | 1.85 (0.85) | ***<0.001*** | 2.18 | 1.89 (0.81) | ***<0.001*** | 2.33 | 1.67 (1.05) | | ***<0.001*** | 1.59 |
| PSS f 20/ ASES f 09 | 1.03 (0.85) | ***<0.001*** | 1.22 | 1.00 (0.82) | ***<0.001*** | 1.22 | 1.20 (1.01) | | ***<0.001*** | 1.18 |
| PSS-Pain | 13.85 (5.67) | ***<0.001*** | 2.44 | 13.75 (5.69) | ***<0.001*** | 2.42 | 14.3 (5.74) | | ***<0.001*** | 2.49 |
| PSS pain at rest | -2.58 (2.35) | ***<0.001*** | -1.10 | -2.47 (2.16) | ***<0.001*** | -1.15 | -3.05 (3.10) | | ***<0.001*** | -0.98 |
| PSS pain normal activities | -4.59 (2.41) | ***<0.001*** | -1.91 | -4.53 (2.41) | ***<0.001*** | -1.88 | -4.90 (2.45) | | ***<0.001*** | -2.00 |
| PSS pain strenuous activities | -6.68 (2.16) | ***<0.001*** | -3.10 | -6.75 (2.13) | ***<0.001*** | -3.16 | -6.35 (2.28) | | ***<0.001*** | -2.79 |
| PSS-Satisfaction | 6.73 (3.30) | ***<0.001*** | 2.04 | 6.80 (3.40) | ***<0.001*** | 2.00 | 6.40 (2.84) | | ***<0.001*** | 2.26 |
| **ASES-Total** | 40.31 (18.48) | ***<0.001*** | 2.18 | 39.34 (17.71) | ***<0.001*** | 2.22 | 44.84 (21.64) | | ***<0.001*** | 2.07 |
| ASES-Function | 23.54 (9.38) | ***<0.001*** | 2.51 | 23.42 (9.18) | ***<0.001*** | 2.55 | 24.09 (10.50) | | ***<0.001*** | 2.29 |
| ASES-Pain | 16.77 (13.40) | ***<0.001*** | 1.25 | 15.91 (13.04) | ***<0.001*** | 1.22 | 20.75 (14.62) | | ***<0.001*** | 1.42 |
| ASES pain today | -3.35 (2.68) | ***<0.001*** | -1.25 | -3.18 (2.61) | ***<0.001*** | -1.22 | -4.15 (2.92) | | ***<0.001*** | -1.42 |
| **SAL** | 0.35 (4.62) | 0.42 | 0.08 | -0.02 (4.43) | 0.96 | 0.00 | 2.10 (5.20) | | 0.087 | 0.40 |
| SAL 1 | 0.12 (1.14) | 0.29 | 0.10 | 0.08 (1.14) | 0.53 | 0.07 | 0.30 (1.13) | | 0.25 | 0.27 |
| SAL 2 | 0.00 (1.30) | 0.99 | 0.00 | -0.09 (1.25) | 0.51 | -0.07 | 0.40 (1.50) | | 0.25 | 0.27 |
| SAL 3 | 0.27 (1.41) | ***0.047*** | 0.19 | 0.20 (1.44) | 0.18 | 0.14 | 0.55 (1.23) | | 0.061 | 0.45 |
| SAL 4 | -0.04 (1.31) | 0.72 | -0.03 | -0.11 (1.29) | 0.42 | -0.08 | 0.25 (1.37) | | 0.43 | 0.18 |
| SAL 5 | 0.02 (1.54) | 0.90 | 0.01 | -0.11 (1.51) | 0.49 | -0.07 | 0.60 (1.60) | | 0.11 | 0.37 |
| **SANE** | 52.68 (26.42) | ***<0.001*** | 1.99 | 53.04 (25.68) | ***<0.001*** | 2.07 | 51.00 (30.27) | | ***<0.001*** | 1.68 |
| **WORC** | - | - | - | - | - | - | - | | - | - |
| WORC 11 | -49.04 (26.96) | ***<0.001*** | -1.82 | -47.91 (27.19) | ***<0.001*** | -1.76 | -54.25 (25.87) | | ***<0.001*** | -2.10 |
| WORC 12 | -57.86 (33.47) | ***<0.001*** | -1.73 | -57.61 (33.63) | ***<0.001*** | -1.71 | -59.00 (33.52) | | ***<0.001*** | -1.76 |
| WORC 13 | -54.64 (30.60) | ***<0.001*** | -1.79 | -54.69 (29.37) | ***<0.001*** | -1.86 | -54.40 (36.61) | | ***<0.001*** | -1.49 |
| WORC 14 | -47.89 (33.16) | ***<0.001*** | -1.44 | -48.17 (32.37) | ***<0.001*** | -1.49 | -46.60 (37.49) | | ***<0.001*** | -1.24 |
| WORC 15 | -58.55 (30.60) | ***<0.001*** | -1.91 | -58.87 (31.32) | ***<0.001*** | -1.88 | -57.05 (27.70) | | ***<0.001*** | -2.06 |
| WORC 16 | -54.10 (29.79) | ***<0.001*** | -1.82 | -54.92 (27.65) | ***<0.001*** | -1.99 | -50.25 (38.85) | | ***<0.001*** | -1.29 |
| WORC 18 | -49.46 (24.04) | ***<0.001*** | -2.06 | -49.48 (22.92) | ***<0.001*** | -2.16 | -49.35 (29.41) | | ***<0.001*** | -1.68 |
| **PROMIS-UE** | 16.68 (8.44) | ***<0.001*** | 1.98 | 16.74 (8.12) | ***<0.001*** | 2.06 | 16.37 (10.00) | | ***<0.001*** | 1.64 |
| promis_pfa29r1 | 1.68 (1.10) | ***<0.001*** | 1.52 | 1.71 (1.09) | ***<0.001*** | 1.57 | 1.55 (1.19) | | ***<0.001*** | 1.30 |
| promis_pfa16r1 | 1.26 (0.95) | ***<0.001*** | 1.32 | 1.26 (0.90) | ***<0.001*** | 1.40 | 1.25 (1.21) | | ***<0.001*** | 1.03 |
| promis_pfb22 | 0.97 (1.04) | ***<0.001*** | 0.94 | 0.90 (0.98) | ***<0.001*** | 0.92 | 1.32 (1.25) | | ***<0.001*** | 1.05 |

*p-value based on paired t-test; SRM = standardized response mean. Note that not all PROM items had n=113 since some items in the PSS function and were not applicable and some PROMIS-UE items were not asked in some patients, as indicated in **Table S1**.

Patients were classified as healed vs not healed at 1-year using observed data or mean of imputed values.

**Table S8.** Paired t-tests of PROM total scores and item level changes from pre-op to 2-year, for healed and not healed subgroups defined by *conventional* criteria at 2-year.

|  | **All (n=113)** | | | **Healed (n=94)** | | | **Not Healed (n=19)** | | |
| --- | --- | --- | --- | --- | --- | --- | --- | --- | --- |
|  | **Change**  **Mean (SD)** | **p-value** | **SRM** | **Change**  **Mean (SD)** | **p-value** | **SRM** | **Change**  **Mean (SD)** | **p-value** | **SRM** |
| **PSS-Total** | 50.56 (16.83) | ***<0.001*** | 3.00 | 50.29 (15.59) | ***<0.001*** | **3.23** | 51.87 (22.48) | ***<0.001*** | **2.31** |
| PSS-Function | 29.39 (10.61) | ***<0.001*** | 2.77 | 29.34 (9.83) | ***<0.001*** | 2.98 | 29.66 (14.16) | ***<0.001*** | 2.09 |
| PSS f 01 | 1.33 (0.83) | ***<0.001*** | 1.60 | 1.33 (0.85) | ***<0.001*** | 1.57 | 1.32 (0.75) | ***<0.001*** | 1.76 |
| PSS f 02/ ASES f 03 | 1.60 (0.95) | ***<0.001*** | 1.68 | 1.60 (0.89) | ***<0.001*** | 1.80 | 1.58 (1.22) | ***<0.001*** | 1.30 |
| PSS f 03/ ASES f 04 | 0.86 (0.86) | ***<0.001*** | 0.99 | 0.84 (0.87) | ***<0.001*** | 0.96 | 0.95 (0.85) | ***<0.001*** | 1.12 |
| PSS f 04 | 1.66 (0.99) | ***<0.001*** | 1.68 | 1.68 (1.03) | ***<0.001*** | 1.63 | 1.58 (0.77) | ***<0.001*** | 2.05 |
| PSS f 05/ ASES f 05 | 1.26 (0.88) | ***<0.001*** | 1.44 | 1.29 (0.83) | ***<0.001*** | 1.54 | 1.11 (1.08) | ***<0.001*** | 1.03 |
| PSS f 06 | 1.37 (0.95) | ***<0.001*** | 1.45 | 1.34 (0.87) | ***<0.001*** | 1.53 | 1.53 (1.26) | ***<0.001*** | 1.21 |
| PSS f 07/ ASES f 01 | 1.17 (0.64) | ***<0.001*** | 1.83 | 1.16 (0.59) | ***<0.001*** | 1.96 | 1.21 (0.86) | ***<0.001*** | 1.42 |
| PSS f 08/ ASES f 02 | 1.69 (0.89) | ***<0.001*** | 1.91 | 1.71 (0.82) | ***<0.001*** | 2.08 | 1.61 (1.20) | ***<0.001*** | 1.35 |
| PSS f 09 | 1.09 (0.74) | ***<0.001*** | 1.47 | 1.10 (0.72) | ***<0.001*** | 1.52 | 1.05 (0.85) | ***<0.001*** | 1.24 |
| PSS f 10 | 1.28 (0.76) | ***<0.001*** | 1.69 | 1.24 (0.73) | ***<0.001*** | 1.71 | 1.47 (0.90) | ***<0.001*** | 1.63 |
| PSS f 11 | 1.12 (0.79) | ***<0.001*** | 1.42 | 1.10 (0.78) | ***<0.001*** | 1.41 | 1.21 (0.86) | ***<0.001*** | 1.42 |
| PSS f 12 | 1.50 (0.88) | ***<0.001*** | 1.71 | 1.47 (0.84) | ***<0.001*** | 1.75 | 1.68 (1.06) | ***<0.001*** | 1.59 |
| PSS f 13 | 1.85 (0.97) | ***<0.001*** | 1.92 | 1.85 (0.94) | ***<0.001*** | 1.97 | 1.84 (1.12) | ***<0.001*** | 1.65 |
| PSS f 14/ ASES f 06 | 1.83 (0.89) | ***<0.001*** | 2.07 | 1.85 (0.84) | ***<0.001*** | 2.20 | 1.74 (1.10) | ***<0.001*** | 1.58 |
| PSS f 15 | 1.81 (0.87) | ***<0.001*** | 2.06 | 1.84 (0.86) | ***<0.001*** | 2.14 | 1.63 (0.96) | ***<0.001*** | 1.71 |
| PSS f 16/ ASES f 07 | 1.89 (0.88) | ***<0.001*** | 2.15 | 1.88 (0.90) | ***<0.001*** | 2.09 | 1.95 (0.78) | ***<0.001*** | 2.50 |
| PSS f 17/ ASES f 10 | 1.71 (0.95) | ***<0.001*** | 1.81 | 1.69 (0.89) | ***<0.001*** | 1.91 | 1.78 (1.22) | ***<0.001*** | 1.46 |
| PSS f 18 | 1.14 (0.65) | ***<0.001*** | 1.75 | 1.16 (0.61) | ***<0.001*** | 1.90 | 1.05 (0.85) | ***<0.001*** | 1.24 |
| PSS f 19/ ASES f 08 | 2.14 (0.81) | ***<0.001*** | 2.66 | 2.11 (0.79) | ***<0.001*** | 2.68 | 2.28 (0.89) | ***<0.001*** | 2.55 |
| PSS f 20/ ASES f 09 | 1.06 (0.81) | ***<0.001*** | 1.30 | 1.03 (0.75) | ***<0.001*** | 1.36 | 1.25 (1.14) | ***0.003*** | 1.10 |
| PSS-Pain | 14.04 (5.96) | ***<0.001*** | 2.35 | 13.84 (5.58) | ***<0.001*** | 2.48 | 15.05 (7.71) | ***<0.001*** | 1.95 |
| PSS pain at rest | -2.50 (2.36) | ***<0.001*** | -1.06 | -2.35 (2.10) | ***<0.001*** | -1.12 | -3.21 (3.33) | ***<0.001*** | -0.97 |
| PSS pain normal activities | -4.68 (2.46) | ***<0.001*** | -1.90 | -4.67 (2.32) | ***<0.001*** | -2.02 | -4.74 (3.16) | ***<0.001*** | -1.50 |
| PSS pain strenuous activities | -6.87 (2.37) | ***<0.001*** | -2.90 | -6.82 (2.35) | ***<0.001*** | -2.91 | -7.11 (2.51) | ***<0.001*** | -2.83 |
| PSS-Satisfaction | 7.12 (3.04) | ***<0.001*** | 2.34 | 7.12 (3.08) | ***<0.001*** | 2.31 | 7.16 (2.89) | ***<0.001*** | 2.48 |
| **ASES-Total** | 42.72 (18.87) | ***<0.001*** | 2.26 | 42.1 (17.45) | ***<0.001*** | **2.41** | 45.8 (25.13) | ***<0.001*** | **1.82** |
| ASES-Function | 25.38 (9.23) | ***<0.001*** | 2.75 | 25.34 (8.50) | ***<0.001*** | 2.98 | 25.54 (12.5) | ***<0.001*** | 2.04 |
| ASES-Pain | 17.35 (13.31) | ***<0.001*** | 1.30 | 16.76 (12.57) | ***<0.001*** | 1.33 | 20.26 (16.62) | ***<0.001*** | 1.22 |
| ASES pain today | -3.47 (2.66) | ***<0.001*** | -1.30 | -3.35 (2.51) | ***<0.001*** | **-1.33** | -4.05 (3.32) | ***<0.001*** | **-1.22** |
| **SAL** | 0.08 (4.22) | 0.84 | 0.02 | -0.33 (4.01) | 0.43 | -0.08 | 2.11 (4.75) | 0.069 | 0.44 |
| SAL 1 | 0.03 (1.23) | 0.82 | 0.02 | -0.07 (1.19) | 0.55 | -0.06 | 0.53 (1.31) | 0.096 | 0.40 |
| SAL 2 | -0.01 (1.18) | 0.94 | -0.01 | -0.09 (1.10) | 0.46 | -0.08 | 0.37 (1.50) | 0.30 | 0.25 |
| SAL 3 | -0.05 (1.47) | 0.70 | -0.04 | -0.07 (1.55) | 0.64 | -0.05 | 0.05 (1.03) | 0.83 | 0.05 |
| SAL 4 | 0.05 (1.25) | 0.65 | 0.04 | -0.02 (1.29) | 0.87 | -0.02 | 0.42 (1.02) | 0.088 | 0.41 |
| SAL 5 | 0.06 (1.38) | 0.63 | 0.04 | -0.07 (1.30) | 0.58 | -0.06 | 0.74 (1.59) | 0.059 | 0.46 |
| **SANE** | 56.20 (26.26) | ***<0.001*** | 2.14 | 56.45 (24.91) | ***<0.001*** | **2.27** | 55.00 (32.93) | ***<0.001*** | **1.67** |
| **WORC** | - | - | - | - | ***-*** | - | - | ***-*** | **-** |
| WORC 11 | -50.99 (28.62) | ***<0.001*** | -1.78 | -51.18 (27.21) | ***<0.001*** | -1.88 | -50.05 (35.63) | ***<0.001*** | -1.40 |
| WORC 12 | -60.96 (33.94) | ***<0.001*** | -1.80 | -62.04 (30.45) | ***<0.001*** | -2.04 | -55.58 (48.41) | ***<0.001*** | -1.15 |
| WORC 13 | -58.85 (28.95) | ***<0.001*** | -2.03 | -59.06 (27.18) | ***<0.001*** | -2.17 | -57.79 (37.39) | ***<0.001*** | -1.55 |
| WORC 14 | -48.15 (35.94) | ***<0.001*** | -1.34 | -48.50 (35.33) | ***<0.001*** | -1.37 | -46.42 (39.82) | ***<0.001*** | -1.17 |
| WORC 15 | -60.92 (28.63) | ***<0.001*** | -2.13 | -61.77 (27.54) | ***<0.001*** | -2.24 | -56.74 (34.07) | ***<0.001*** | -1.67 |
| WORC 16 | -56.37 (28.78) | ***<0.001*** | -1.96 | -57.61 (26.36) | ***<0.001*** | -2.19 | -50.26 (38.91) | ***<0.001*** | -1.29 |
| WORC 18 | -51.00 (23.56) | ***<0.001*** | -2.17 | -51.26 (21.65) | ***<0.001*** | -2.37 | -49.74 (32.07) | ***<0.001*** | -1.55 |
| **PROMIS-UE** | 18.44 (8.35) | ***<0.001*** | 2.21 | 18.05 (8.02) | ***<0.001*** | **2.25** | 20.37 (9.83) | ***<0.001*** | **2.07** |
| promis_pfa29r1 | 1.76 (1.16) | ***<0.001*** | 1.52 | 1.69 (1.15) | ***<0.001*** | 1.46 | 2.11 (1.15) | ***<0.001*** | 1.83 |
| promis_pfa16r1 | 1.26 (0.90) | ***<0.001*** | 1.39 | 1.23 (0.85) | ***<0.001*** | 1.46 | 1.37 (1.16) | ***<0.001*** | 1.17 |
| promis_pfb22 | 0.95 (0.98) | ***<0.001*** | 0.97 | 0.86 (0.91) | ***<0.001*** | 0.95 | 1.39 (1.20) | ***<0.001*** | 1.16 |

*p-value based on paired t-test; SRM = standardized response mean. Note that not all PROM items had n=113 since some items in the PSS function and were not applicable and some PROMIS-UE items were not asked in some patients, as indicated in **Table S1**.

Patients were classified as healed vs not healed at 2-year using observed data or mean of imputed values.

**Table S9.** Paired t-tests of PROM total scores and item level changes from pre-op to 6-month, for healed and not healed subgroups defined by *stringent* criteria at 6-month.

|  | **Healed (n=58)** | | | **Not Healed (n=18)** | | |
| --- | --- | --- | --- | --- | --- | --- |
|  | **Change**  **Mean (SD)** | **p-value** | **SRM** | **Change**  **Mean (SD)** | **p-value** | **SRM** |
| **PSS-Total** | 39.84 (16.71) | ***<0.001*** | 2.38 | 45.17 (18.90) | ***<0.001*** | 2.39 |
| PSS-Function | 21.75 (10.51) | ***<0.001*** | 2.07 | 26.67 (11.97) | ***<0.001*** | 2.23 |
| PSS f 01 | 0.83 (0.88) | ***<0.001*** | 0.94 | 1.17 (0.92) | ***<0.001*** | 1.26 |
| PSS f 02/ ASES f 03 | 0.84 (0.95) | ***<0.001*** | 0.89 | 1.28 (1.13) | ***<0.001*** | 1.13 |
| PSS f 03/ ASES f 04 | 0.76 (0.76) | ***<0.001*** | 1.00 | 0.83 (0.71) | ***<0.001*** | 1.18 |
| PSS f 04 | 1.09 (1.03) | ***<0.001*** | 1.05 | 1.28 (0.83) | ***<0.001*** | 1.55 |
| PSS f 05/ ASES f 05 | 1.05 (0.80) | ***<0.001*** | 1.32 | 1.11 (0.96) | ***<0.001*** | 1.15 |
| PSS f 06 | 1.02 (1.10) | ***<0.001*** | 0.92 | 1.22 (1.35) | ***0.001*** | 0.90 |
| PSS f 07/ ASES f 01 | 0.88 (0.68) | ***<0.001*** | 1.30 | 1.11 (0.83) | ***<0.001*** | 1.33 |
| PSS f 08/ ASES f 02 | 1.22 (1.08) | ***<0.001*** | 1.14 | 1.22 (1.06) | ***<0.001*** | 1.15 |
| PSS f 09 | 0.86 (0.78) | ***<0.001*** | 1.10 | 0.94 (0.80) | ***<0.001*** | 1.18 |
| PSS f 10 | 0.97 (0.82) | ***<0.001*** | 1.18 | 1.44 (0.70) | ***<0.001*** | 2.05 |
| PSS f 11 | 0.86 (0.83) | ***<0.001*** | 1.03 | 1.33 (0.77) | ***<0.001*** | 1.74 |
| PSS f 12 | 1.34 (0.93) | ***<0.001*** | 1.45 | 1.50 (0.92) | ***<0.001*** | 1.62 |
| PSS f 13 | 1.36 (1.05) | ***<0.001*** | 1.29 | 1.67 (0.91) | ***<0.001*** | 1.84 |
| PSS f 14/ ASES f 06 | 1.36 (0.95) | ***<0.001*** | 1.43 | 1.61 (0.98) | ***<0.001*** | 1.65 |
| PSS f 15 | 1.57 (1.06) | ***<0.001*** | 1.48 | 1.61 (1.04) | ***<0.001*** | 1.55 |
| PSS f 16/ ASES f 07 | 1.33 (0.98) | ***<0.001*** | 1.35 | 1.50 (0.71) | ***<0.001*** | 2.12 |
| PSS f 17/ ASES f 10 | 1.09 (0.97) | ***<0.001*** | 1.13 | 1.71 (0.85) | ***<0.001*** | 2.01 |
| PSS f 18 | 0.98 (0.69) | ***<0.001*** | 1.43 | 1.11 (0.83) | ***<0.001*** | 1.33 |
| PSS f 19/ ASES f 08 | 1.36 (0.82) | ***<0.001*** | 1.66 | 1.73 (1.01) | ***<0.001*** | 1.71 |
| PSS f 20/ ASES f 09 | 0.82 (0.90) | ***<0.001*** | 0.91 | 0.93 (0.92) | ***0.002*** | 1.01 |
| PSS-Pain | 12.24 (5.47) | ***<0.001*** | 2.24 | 12.44 (6.13) | ***<0.001*** | 2.03 |
| PSS pain at rest | -2.41 (2.35) | ***<0.001*** | -1.03 | -2.89 (3.23) | ***0.002*** | -0.89 |
| PSS pain normal activities | -4.03 (2.46) | ***<0.001*** | -1.64 | -4.17 (2.50) | ***<0.001*** | -1.66 |
| PSS pain strenuous activities | -5.79 (1.99) | ***<0.001*** | -2.91 | -5.39 (2.68) | ***<0.001*** | -2.01 |
| PSS-Satisfaction | 5.84 (3.07) | ***<0.001*** | 1.90 | 6.06 (2.96) | ***<0.001*** | 2.05 |
| **ASES-Total** | 34.15 (19.72) | ***<0.001*** | 1.73 | 37.82 (24.30) | ***<0.001*** | 1.56 |
| ASES-Function | 18.12 (9.15) | ***<0.001*** | 1.98 | 22.27 (10.35) | ***<0.001*** | 2.15 |
| ASES-Pain | 16.03 (14.47) | ***<0.001*** | 1.11 | 15.56 (15.52) | ***0.001*** | 1.00 |
| ASES pain today | -3.21 (2.89) | ***<0.001*** | -1.11 | -3.11 (3.10) | ***0.001*** | -1.00 |
| **SAL** | -0.43 (4.46) | 0.46 | -0.10 | 3.00 (5.51) | ***0.034*** | 0.54 |
| SAL 1 | -0.31 (1.26) | 0.066 | -0.25 | 0.39 (1.54) | 0.30 | 0.25 |
| SAL 2 | -0.29 (1.39) | 0.11 | -0.21 | 0.83 (2.04) | 0.10 | 0.41 |
| SAL 3 | 0.36 (1.39) | 0.051 | 0.26 | 0.67 (1.33) | ***0.048*** | 0.50 |
| SAL 4 | -0.09 (1.27) | 0.61 | -0.07 | 0.50 (1.42) | 0.16 | 0.35 |
| SAL 5 | -0.10 (1.24) | 0.53 | -0.08 | 0.61 (1.82) | 0.17 | 0.34 |
| **SANE** | 44.09 (24.85) | ***<0.001*** | 1.77 | 54.00 (26.37) | ***<0.001*** | 2.05 |
| **WORC** | - | ***-*** | - | - | ***-*** | **-** |
| WORC 11 | -37.64 (26.36) | ***<0.001*** | -1.43 | -45.94 (41.69) | ***<0.001*** | -1.10 |
| WORC 12 | -45.84 (31.43) | ***<0.001*** | -1.46 | -50.94 (45.57) | ***<0.001*** | -1.12 |
| WORC 13 | -36.41 (32.17) | ***<0.001*** | -1.13 | -49.94 (39.49) | ***<0.001*** | -1.26 |
| WORC 14 | -33.90 (30.81) | ***<0.001*** | -1.10 | -42.39 (42.19) | ***0.001*** | -1.00 |
| WORC 15 | -50.03 (29.67) | ***<0.001*** | -1.69 | -43.89 (44.59) | ***0.001*** | -0.98 |
| WORC 16 | -49.59 (27.23) | ***<0.001*** | -1.82 | -38.78 (48.76) | ***0.004*** | -0.80 |
| WORC 18 | -47.69 (21.06) | ***<0.001*** | -2.26 | -42.94 (33.40) | ***<0.001*** | -1.29 |
| **PROMIS-UE** | 11.17 (7.25) | ***<0.001*** | 1.54 | 14.07 (9.75) | ***<0.001*** | 1.44 |
| promis_pfa29r1 | 1.40 (1.01) | ***<0.001*** | 1.39 | 1.44 (1.46) | ***<0.001*** | 0.99 |
| promis_pfa16r1 | 1.05 (0.91) | ***<0.001*** | 1.16 | 1.11 (1.02) | ***<0.001*** | 1.09 |
| promis_pfb22 | 0.78 (0.88) | ***<0.001*** | 0.88 | 1.38 (1.15) | ***<0.001*** | 1.20 |

*p-value based on paired t-test; SRM = standardized response mean.

Patients were classified as healed vs not healed at 6-month using observed data or mean of imputed values.

**Table S10.** Paired t-tests of PROM total scores and item level changes from pre-op to 1-year, for healed and not healed subgroups defined by *stringent* criteria at 1-year.

|  | **Healed (n=47)** | | | **Not Healed (n=15)** | | |
| --- | --- | --- | --- | --- | --- | --- |
|  | **Change**  **Mean (SD)** | **p-value** | **SRM** | **Change**  **Mean (SD)** | **p-value** | **SRM** |
| **PSS-Total** | 48.32 (16.35) | ***<0.001*** | 2.96 | 53.75 (18.45) | ***<0.001*** | 2.91 |
| PSS-Function | 28.11 (10.38) | ***<0.001*** | 2.71 | 31.95 (11.45) | ***<0.001*** | 2.79 |
| PSS f 01 | 1.19 (0.88) | ***<0.001*** | 1.36 | 1.40 (0.74) | ***<0.001*** | 1.90 |
| PSS f 02/ ASES f 03 | 1.45 (0.97) | ***<0.001*** | 1.49 | 1.62 (1.04) | ***<0.001*** | 1.55 |
| PSS f 03/ ASES f 04 | 0.93 (0.88) | ***<0.001*** | 1.06 | 1.00 (0.88) | ***0.001*** | 1.14 |
| PSS f 04 | 1.55 (0.97) | ***<0.001*** | 1.60 | 1.60 (0.83) | ***<0.001*** | 1.93 |
| PSS f 05/ ASES f 05 | 1.24 (0.80) | ***<0.001*** | 1.55 | 1.36 (0.84) | ***<0.001*** | 1.61 |
| PSS f 06 | 1.28 (0.95) | ***<0.001*** | 1.35 | 1.80 (1.01) | ***<0.001*** | 1.77 |
| PSS f 07/ ASES f 01 | 1.06 (0.57) | ***<0.001*** | 1.87 | 1.40 (0.83) | ***<0.001*** | 1.69 |
| PSS f 08/ ASES f 02 | 1.55 (1.00) | ***<0.001*** | 1.56 | 1.64 (0.84) | ***<0.001*** | 1.95 |
| PSS f 09 | 1.13 (0.80) | ***<0.001*** | 1.41 | 1.07 (0.88) | ***<0.001*** | 1.21 |
| PSS f 10 | 1.17 (0.87) | ***<0.001*** | 1.35 | 1.33 (0.90) | ***<0.001*** | 1.48 |
| PSS f 11 | 1.02 (0.87) | ***<0.001*** | 1.17 | 1.47 (0.74) | ***<0.001*** | 1.97 |
| PSS f 12 | 1.60 (0.85) | ***<0.001*** | 1.87 | 1.93 (0.80) | ***<0.001*** | 2.42 |
| PSS f 13 | 1.81 (1.01) | ***<0.001*** | 1.78 | 2.07 (0.80) | ***<0.001*** | 2.59 |
| PSS f 14/ ASES f 06 | 1.81 (0.85) | ***<0.001*** | 2.13 | 2.00 (0.93) | ***<0.001*** | 2.16 |
| PSS f 15 | 1.85 (0.88) | ***<0.001*** | 2.09 | 1.87 (1.06) | ***<0.001*** | 1.76 |
| PSS f 16/ ASES f 07 | 1.70 (1.00) | ***<0.001*** | 1.71 | 1.80 (0.77) | ***<0.001*** | 2.32 |
| PSS f 17/ ASES f 10 | 1.64 (0.86) | ***<0.001*** | 1.92 | 2.07 (0.73) | ***<0.001*** | 2.84 |
| PSS f 18 | 1.13 (0.68) | ***<0.001*** | 1.66 | 1.27 (0.80) | ***<0.001*** | 1.59 |
| PSS f 19/ ASES f 08 | 1.93 (0.86) | ***<0.001*** | 2.25 | 1.90 (1.10) | ***<0.001*** | 1.73 |
| PSS f 20/ ASES f 09 | 1.08 (0.81) | ***<0.001*** | 1.33 | 1.40 (1.08) | ***0.003*** | 1.30 |
| PSS-Pain | 13.53 (5.71) | ***<0.001*** | 2.37 | 15.13 (5.64) | ***<0.001*** | 2.68 |
| PSS pain at rest | -2.40 (2.20) | ***<0.001*** | -1.09 | -3.47 (3.25) | ***0.001*** | -1.07 |
| PSS pain normal activities | -4.34 (2.36) | ***<0.001*** | -1.84 | -5.20 (2.48) | ***<0.001*** | -2.09 |
| PSS pain strenuous activities | -6.79 (2.11) | ***<0.001*** | -3.22 | -6.47 (2.42) | ***<0.001*** | -2.68 |
| PSS-Satisfaction | 6.68 (3.18) | ***<0.001*** | 2.10 | 6.67 (2.77) | ***<0.001*** | 2.41 |
| **ASES-Total** | 40.12 (18.92) | ***<0.001*** | 2.12 | 49.56 (20.41) | ***<0.001*** | 2.43 |
| ASES-Function | 23.95 (8.87) | ***<0.001*** | 2.70 | 26.89 (9.75) | ***<0.001*** | 2.76 |
| ASES-Pain | 16.17 (13.92) | ***<0.001*** | 1.16 | 22.67 (14.74) | ***<0.001*** | 1.54 |
| ASES pain today | -3.23 (2.78) | ***<0.001*** | -1.16 | -4.53 (2.95) | ***<0.001*** | -1.54 |
| **SAL** | -0.81 (4.03) | 0.18 | -0.20 | 3.27 (5.39) | ***0.034*** | 0.61 |
| SAL 1 | -0.09 (1.18) | 0.62 | -0.07 | 0.40 (1.30) | 0.25 | 0.31 |
| SAL 2 | -0.23 (1.07) | 0.14 | -0.22 | 0.73 (1.53) | 0.085 | 0.48 |
| SAL 3 | 0.15 (1.44) | 0.48 | 0.10 | 0.73 (1.39) | 0.060 | 0.53 |
| SAL 4 | -0.43 (1.35) | ***0.036*** | -0.32 | 0.60 (1.12) | 0.057 | 0.54 |
| SAL 5 | -0.21 (1.46) | 0.32 | -0.15 | 0.80 (1.61) | 0.075 | 0.50 |
| **SANE** | 54.53 (24.19) | ***<0.001*** | 2.25 | 57.00 (32.12) | ***<0.001*** | 1.77 |
| **WORC** | - | ***-*** | - | - | ***-*** | **-** |
| WORC 11 | -47.51 (25.65) | ***<0.001*** | -1.85 | -58.40 (27.40) | ***<0.001*** | -2.13 |
| WORC 12 | -58.79 (29.46) | ***<0.001*** | -2.00 | -64.00 (35.56) | ***<0.001*** | -1.80 |
| WORC 13 | -53.55 (29.84) | ***<0.001*** | -1.79 | -59.87 (37.02) | ***<0.001*** | -1.62 |
| WORC 14 | -49.74 (31.92) | ***<0.001*** | -1.56 | -56.33 (33.93) | ***<0.001*** | -1.66 |
| WORC 15 | -59.70 (29.77) | ***<0.001*** | -2.01 | -57.80 (25.67) | ***<0.001*** | -2.25 |
| WORC 16 | -59.15 (24.46) | ***<0.001*** | -2.42 | -50.93 (39.19) | ***<0.001*** | -1.30 |
| WORC 18 | -52.19 (18.29) | ***<0.001*** | -2.85 | -50.53 (29.15) | ***<0.001*** | -1.73 |
| **PROMIS-UE** | 15.45 (7.79) | ***<0.001*** | 1.98 | 18.39 (10.17) | ***<0.001*** | 1.81 |
| promis_pfa29r1 | 1.74 (1.09) | ***<0.001*** | 1.60 | 1.80 (1.21) | ***<0.001*** | 1.49 |
| promis_pfa16r1 | 1.15 (0.86) | ***<0.001*** | 1.34 | 1.47 (1.25) | ***<0.001*** | 1.18 |
| promis_pfb22 | 0.85 (0.93) | ***<0.001*** | 0.91 | 1.53 (1.25) | ***<0.001*** | 1.23 |

*p-value based on paired t-test; SRM = standardized response mean.

Patients were classified as healed vs not healed at 1-year using observed data or mean of imputed values.

**Table S11.** Paired t-tests of PROM total scores and item level changes from pre-op to 2-year for healed and not healed subgroups defined by *stringent* criteria at 2-year.

|  | **Healed (n=41)** | | | **Not Healed (n=15)** | | |
| --- | --- | --- | --- | --- | --- | --- |
|  | **Change**  **Mean (SD)** | **p-value** | **SRM** | **Change**  **Mean (SD)** | **p-value** | **SRM** |
| **PSS-Total** | 49.26 (14.82) | ***<0.001*** | 3.32 | 49.71 (23.57) | ***<0.001*** | 2.11 |
| PSS-Function | 28.92 (9.13) | ***<0.001*** | 3.17 | 28.78 (15.16) | ***<0.001*** | 1.90 |
| PSS f 01 | 1.24 (0.73) | ***<0.001*** | 1.69 | 1.33 (0.72) | ***<0.001*** | 1.84 |
| PSS f 02/ ASES f 03 | 1.62 (0.88) | ***<0.001*** | 1.84 | 1.53 (1.30) | ***<0.001*** | 1.18 |
| PSS f 03/ ASES f 04 | 0.83 (0.92) | ***<0.001*** | 0.90 | 1.00 (0.93) | ***0.001*** | 1.08 |
| PSS f 04 | 1.73 (0.95) | ***<0.001*** | 1.82 | 1.40 (0.74) | ***<0.001*** | 1.90 |
| PSS f 05/ ASES f 05 | 1.18 (0.76) | ***<0.001*** | 1.56 | 1.13 (1.13) | ***0.002*** | 1.01 |
| PSS f 06 | 1.37 (0.86) | ***<0.001*** | 1.59 | 1.47 (1.30) | ***<0.001*** | 1.13 |
| PSS f 07/ ASES f 01 | 1.10 (0.62) | ***<0.001*** | 1.76 | 1.13 (0.92) | ***<0.001*** | 1.24 |
| PSS f 08/ ASES f 02 | 1.70 (0.79) | ***<0.001*** | 2.15 | 1.43 (1.22) | ***<0.001*** | 1.17 |
| PSS f 09 | 1.15 (0.85) | ***<0.001*** | 1.34 | 0.93 (0.88) | ***0.001*** | 1.06 |
| PSS f 10 | 1.10 (0.77) | ***<0.001*** | 1.43 | 1.33 (0.90) | ***<0.001*** | 1.48 |
| PSS f 11 | 0.98 (0.79) | ***<0.001*** | 1.23 | 1.33 (0.82) | ***<0.001*** | 1.63 |
| PSS f 12 | 1.54 (0.81) | ***<0.001*** | 1.90 | 1.60 (0.99) | ***<0.001*** | 1.62 |
| PSS f 13 | 1.80 (1.03) | ***<0.001*** | 1.75 | 1.87 (1.06) | ***<0.001*** | 1.76 |
| PSS f 14/ ASES f 06 | 1.88 (0.78) | ***<0.001*** | 2.41 | 1.53 (1.06) | ***<0.001*** | 1.45 |
| PSS f 15 | 1.93 (0.79) | ***<0.001*** | 2.45 | 1.47 (0.92) | ***<0.001*** | 1.60 |
| PSS f 16/ ASES f 07 | 1.85 (0.94) | ***<0.001*** | 1.98 | 1.80 (0.77) | ***<0.001*** | 2.32 |
| PSS f 17/ ASES f 10 | 1.72 (0.94) | ***<0.001*** | 1.82 | 1.93 (1.21) | ***<0.001*** | 1.60 |
| PSS f 18 | 1.15 (0.57) | ***<0.001*** | 2.00 | 1.13 (0.92) | ***<0.001*** | 1.24 |
| PSS f 19/ ASES f 08 | 2.03 (0.82) | ***<0.001*** | 2.47 | 2.21 (0.98) | ***<0.001*** | 2.27 |
| PSS f 20/ ASES f 09 | 0.97 (0.77) | ***<0.001*** | 1.26 | 1.40 (1.17) | ***0.004*** | 1.19 |
| PSS-Pain | 13.00 (5.80) | ***<0.001*** | 2.24 | 14.27 (7.67) | ***<0.001*** | 1.86 |
| PSS pain at rest | -2.20 (2.15) | ***<0.001*** | -1.02 | -3.07 (3.43) | ***0.004*** | -0.89 |
| PSS pain normal activities | -4.22 (2.50) | ***<0.001*** | -1.69 | -4.47 (3.18) | ***<0.001*** | -1.40 |
| PSS pain strenuous activities | -6.59 (2.43) | ***<0.001*** | -2.71 | -6.73 (2.60) | ***<0.001*** | -2.59 |
| PSS-Satisfaction | 7.34 (2.48) | ***<0.001*** | 2.97 | 6.67 (3.04) | ***<0.001*** | 2.19 |
| **ASES-Total** | 39.99 (17.79) | ***<0.001*** | 2.25 | 45.58 (25.39) | ***<0.001*** | 1.80 |
| ASES-Function | 24.87 (7.59) | ***<0.001*** | 3.28 | 24.92 (13.63) | ***<0.001*** | 1.83 |
| ASES-Pain | 15.12 (13.62) | ***<0.001*** | 1.11 | 20.67 (15.91) | ***<0.001*** | 1.30 |
| ASES pain today | -3.02 (2.72) | ***<0.001*** | -1.11 | -4.13 (3.18) | ***<0.001*** | -1.30 |
| **SAL** | -0.90 (3.87) | 0.14 | -0.23 | 2.80 (5.03) | ***0.049*** | 0.56 |
| SAL 1 | -0.15 (1.26) | 0.46 | -0.12 | 0.67 (1.40) | 0.086 | 0.48 |
| SAL 2 | -0.17 (0.86) | 0.21 | -0.20 | 0.47 (1.64) | 0.29 | 0.28 |
| SAL 3 | -0.20 (1.60) | 0.44 | -0.12 | 0.33 (0.90) | 0.17 | 0.37 |
| SAL 4 | -0.32 (1.13) | 0.079 | -0.28 | 0.40 (1.12) | 0.19 | 0.36 |
| SAL 5 | -0.07 (1.35) | 0.73 | -0.05 | 0.93 (1.71) | 0.053 | 0.55 |
| **SANE** | 58.88 (22.52) | ***<0.001*** | 2.61 | 52.73 (35.51) | ***<0.001*** | 1.48 |
| **WORC** | - | ***-*** | - | - | ***-*** | **-** |
| WORC 11 | -51.90 (24.36) | ***<0.001*** | -2.13 | -47.20 (38.05) | ***<0.001*** | -1.24 |
| WORC 12 | -64.39 (24.29) | ***<0.001*** | -2.65 | -47.73 (51.39) | ***0.003*** | -0.93 |
| WORC 13 | -60.32 (25.93) | ***<0.001*** | -2.33 | -52.80 (40.84) | ***<0.001*** | -1.29 |
| WORC 14 | -45.63 (32.26) | ***<0.001*** | -1.41 | -43.80 (42.12) | ***0.001*** | -1.04 |
| WORC 15 | -60.98 (26.95) | ***<0.001*** | -2.26 | -55.73 (36.47) | ***<0.001*** | -1.53 |
| WORC 16 | -57.80 (20.71) | ***<0.001*** | -2.79 | -51.53 (41.69) | ***<0.001*** | -1.24 |
| WORC 18 | -53.83 (17.91) | ***<0.001*** | -3.01 | -49.60 (32.90) | ***<0.001*** | -1.51 |
| **PROMIS-UE** | 17.44 (6.68) | ***<0.001*** | 2.61 | 19.43 (10.29) | ***<0.001*** | 1.89 |
| promis_pfa29r1 | 1.8 (1.08) | ***<0.001*** | 1.68 | 2.13 (1.19) | ***<0.001*** | 1.80 |
| promis_pfa16r1 | 1.15 (0.79) | ***<0.001*** | 1.45 | 1.27 (1.16) | ***<0.001*** | 1.09 |
| promis_pfb22 | 0.78 (0.88) | ***<0.001*** | 0.89 | 1.27 (1.28) | ***0.002*** | 0.99 |

*p-value based on paired t-test; SRM = standardized response mean.

Patients were classified as healed vs not healed at 2-year using observed data or mean of imputed values.
